# Efficacy of CBP/p300 Dual Inhibitors against Derepression of KREMEN2 in cBAF-Deficient Cancers

**DOI:** 10.1158/2767-9764.CRC-24-0484

**Published:** 2025-01-06

**Authors:** Mariko Sasaki, Daiki Kato, Hiroshi Yoshida, Takafumi Shimizu, Hideaki Ogiwara

**Affiliations:** 1 Division of Cancer Therapeutics, National Cancer Center Research Institute, Tokyo, Japan.; 2 Cancer Research Unit, Sumitomo Pharma Co., Ltd, Osaka, Japan.; 3 Department of Diagnostic Pathology, National Cancer Center Hospital, Tokyo, Japan.

## Abstract

The SWItch/Sucrose Non-Fermentable (SWI/SNF) chromatin remodeling complex is divided into three subcomplexes: cBAF, PBAF, and ncBAF. Constituent genes (e.g., *SMARCB1*, *SMARCA4*, *SMARCA2*, and *SS18*) of the SWI/SNF complex often harbor genetic abnormalities in various cancers. Previously, we found that histone acetyltransferases CBP/p300 dual inhibitors could be promising treatments for SMARCB1-deficient cancers. We show that treatment with CBP/p300 dual inhibitors causes synthetic lethality in cBAF-deficient cancers such as SMARCA4/SMARCA2-deficient and SS18–SSX fusion cancers. Given the sensitivity to CBP/p300 dual inhibitors and their commonality with SWI/SNF subcomplexes containing each subunit, CBP/p300 dual inhibitors could be promising treatments for cancers harboring abnormalities in constituent genes included in the entire cBAF subcomplex. Because SMARCA4/SMARCA2-deficient and SS18–SSX fusion cancer cells depend on transcriptional upregulation of *KREMEN2* due to SMARCA4/SMARCA2 deficiency and SS18–SSX fusion, we clarified that synthetic lethality is induced by repressing expression of *KREMEN2* by simultaneous inhibition of CBP/p300. In addition, simultaneous inhibition of CBP/p300 led to transcriptional downregulation of *KREMEN2*, followed by apoptosis induction via *KREMEN1*. Furthermore, treatment with CBP/p300 dual inhibitor suppressed the growth of xenografts derived from SMARCA4/SMARCA2-deficient and SS18–SSX fusion cancer cells, resulting from repression of *KREMEN2* and induction of apoptosis. Thus, CBP/p300 dual inhibitors could be promising for SMARCA4/SMARCA2-deficient lung cancer and SS18–SSX fusion synovial sarcoma, which are entirely deficient in the cBAF complex.

**Significance:**

In this study, we clarified that the cBAF subcomplex is deficient in the SWI/SNF complex, resulting in dependency on the CBP/p300 paralog pair. Simultaneous inhibitors of the CBP/p300 paralog pair show promise for cBAF-deficient lung cancer, as well as rare cancers such as malignant rhabdoid tumors, epithelioid sarcomas, and synovial sarcomas.

## Introduction

Cancer genomic medicine, a form of cancer treatment that aims to address genetic mutations within cancerous cells specifically, is expected to advance through improvements in multigene panel testing (1). Cancer genomic medicine is utilized primarily for cancers exhibiting gain-of-function mutations in oncogenes (2). Although there are loss-of-function (LOF) mutations in tumor suppressor genes, these genes cannot be targeted for therapy. Synthetic lethality refers to cell death triggered by simultaneous inhibition of two genes rather than just one gene (3). Currently, synthetic lethal agents that focus on a synthetic lethal factor in cancers displaying LOF mutations are a treatment option for cancer.

Mutations in genes that encode subunits of the SWI/SNF (SWItch/Sucrose Non-Fermentable) chromatin remodeling complex are found in around 20% of all cancer cases (4, 5). This complex comprises approximately 15 subunits and is categorized into three separate subcomplexes: the BRG1/BRM-associated factor (BAF) subcomplex, the polybromo-associated BAF (PBAF) subcomplex, and the noncanonical BAF (ncBAF) subcomplex (6). Many SWI/SNF-related genes are linked to LOF-mediated genetic changes in cancer cells, leading researchers to focus on developing therapies based on synthetic lethality.

The SWI/SNF complex plays a vital role in regulating cellular functions such as transcription by modulating the chromatin structure via regulating genes related to various chromatin-regulating factors (3). The balance between transcriptional promotion and suppression is disrupted in cancers deficient in the SWI/SNF complex, making them vulnerable. Most notably, rhabdoid tumors and epithelioid sarcomas lack SMARCB1 (7, 8). Other SWI/SNF complex components are also deficient in various cancers (3). In particular, SMARCA4, ARID1A, and PBRM1 are lacking in 10% of lung cancer cases, 50% of ovarian clear cell carcinoma cases, and 40% of clear cell renal cell carcinoma cases, respectively (9). SMARCA4 is a component of all cBAFs, PBAFs, and ncBAFs. ARID1A is a component of the cBAF complex, and PBRM1 is a component of PBAF. SMARCA2 is included in cBAF, PBAF, and ncBAF and is mutually exclusively with SMARCA4 (6). Ten percent of lung cancer and most of small cell carcinoma of the ovary, hypercalcemic type (SCCOHT), are deficient in both SMARCA4 and SMARCA2 (10, 11). An in-frame fusion of the SS18 subunit of the SWI/SNF chromatin remodeling complex with a member of the SSX family defines synovial sarcoma. This process results in substituting the final eight amino acids of SS18 with the C-terminal 78 amino acids of SSX1, SSX2, or SSX4 (12, 13).

CBP (*CREBBP*) and p300 (*EP300*) are paralog proteins that work together to acetylate histone H3K27, thereby facilitating transcription by opening the chromatin structure and recruiting the RNA polymerase II machinery and other transcriptional regulators (14–16). Targeting the catalytic histone acetylation domain of CBP/p300 with small-molecule inhibitors such as CP-C27 and A-485 has shown promise as a cancer therapy (17–19). These inhibitors selectively and simultaneously target CBP and its paralog p300 because both of them have similar structures.

Recently, we devised the synthetic lethal target screening method based on “the simultaneous inhibition of a paralog pair as a synthetic lethal target” concept and showed that screening for paralog pairs is a viable approach to identifying innovative synthetic lethal targets (19). By simultaneously inhibiting a paralog pair, it may be possible to develop therapies that effectively target two proteins using a single agent. Indeed, we identified promising simultaneous inhibitors of CBP/p300 in SMARCB1-deficient cancers (19). Based on a search for downstream factors that determine synthetic lethality due to simultaneous inhibition of CBP/p300 in SMARCB1-deficient cells, we identified the *KREMEN2* gene as the only gene upregulated specifically in SMARCB1-deficient cells (19). Furthermore, we proposed the following molecular mechanism underlying synthetic lethality induced by simultaneous inhibition of CBP/p300 in SMARCB1-deficient cancers: in SMARCB1-proficient cells, the SWI/SNF complex containing SMARCB1 represses transcription of KREMEN2. By contrast, the absence of SMARCB1 promotes KREMEN2 expression through the collaborative action of CBP and p300, leading to the inhibition of KREMEN1 via homodimerization. Ultimately, this cascade activates antiapoptotic signaling pathways. Reducing KREMEN2 levels by targeting CBP/p300 induces monomerization of KREMEN1, thereby triggering apoptotic cell death by blocking antiapoptotic signaling pathways (19). Therefore, this study examined the potential for expansion and adaptation of simultaneous CBP/p300 inhibitors for use against cancers with SS18–SSX fusion and the majority of SMARCA4/SMARCA2-deficient in addition to SMARCB1 deficient, which was reported previously (19).

## Materials and Methods

### Animal ethics statement

The mouse experiments were approved by the National Cancer Center Animal Ethical Committee and the Institutional Animal Care and Use Committee of Sumitomo Pharma Co. Ltd (certificate protocol numbers T19-013-M06 and AN13842-B00/AN14078-B00, respectively). They were performed in accordance with the Act on Welfare and Management of Animals. The experiments were carried out according to the Standards for Care and Keeping and Reducing Pain of Laboratory Animals. Mice were checked for clinical indications, tumor size, and body weight as specified in the experimental licenses. Mice were killed before reaching the approved humane endpoints of either a tumor size of 2,000 mm^3^ or a body weight loss of 20% or whenever they showed apparent clinical signs of pain. The maximum tumor size/burden was never exceeded in any of the experiments.

### Cell lines

Cells were maintained at 37°C in a humidified incubator containing 5% CO_2_. The culture medium comprised DMEM/F-12 (Wako, 048-29785) supplemented with 10% FBS (Gibco/Life Technologies), 10% GlutaMAX supplement (Gibco, 41550021), and 100 U/mL penicillin/100 μg/mL streptomycin (Wako, 168-23191). The H358 (CRL-5807, RRID: CVCL_1559), H2009 (CRL-5911, RRID: CVCL_1514), H1048 (CRL-5853, RRID: CVCL_1453), H2228 (CRL-5935, RRID: CVCL_1543), 786-O (CRL-1932, RRID: CVCL_1051), ES-2 (CRL-1978, RRID: CVCL_3509), A549 (CCL-185, RRID: CVCL_0023), H1299 (CRL-5803, RRID: CVCL_0060), H1819 (CRL-5897, RRID: CVCL_1497), H1650 (CRL-5883, RRID: CVCL_1483), A-427 (HTB-53, RRID: CVCL_1055), Caki-2 (HTB-47, RRID: CVCL_0235), DMS114 (CRL-2066, RRID: CVCL_1174), TOV112D (CRL-11731, RRID: CVCL_3612), and TOV-21G (CRL-11730, RRID: CVCL_3613) cell lines were obtained from the ATCC. The OVISE (JCRB1043, RRID: CVCL_3116), OVTOKO (JCRB1048, RRID: CVCL_3117), RMG-I (JCRB0172, RRID: CVCL_1662), RMG-V (JCRB1729, RRID: CVCL_M716), KMRC-1 (JCRB1010, RRID: CVCL_2983), SW-13 (JCRB9069, RRID: CVCL_0542), and SBC-5 (JCRB0819, RRID: CVCL_1679) cell lines were obtained from the Japanese Collection of Research Bioresources Cell Bank. The Aska-SS (RCB3576, RRID: CVCL_6C43), HS-SY-II (RCB2231, RRID: CVCL_8719), OS-RC-2 (RCB0735, RRID: CVCL_1626), and Yamato-SS (RCB3577, RRID: CVCL_6C44) cell lines were obtained from the Riken Cell Bank). The RCC-FG2 (CLS300249, RRID: CVCL_5873) cell line was obtained from Cell Lines Service. Dr. Shinya Tanaka kindly provided the Fuji (RRID: CVCL_D880) cell line (20). The 786-O, A-427, A549, DMS114, ES-2, H1299, H1650, H2228, H358, TOV112D, H1819, and SW-13 cell lines were authenticated in 2020 using the PowerPlex 16 STR System (Promega). Although other cell lines are not authenticated, all were used for functional experiments after less than 2 months of passage post-receipt. All cell lines tested in 2023 were harmful to *Mycoplasma* by MycoAlert (Lonza, LT07-318). No sex- and gender-based analyses were performed because the study focused on genetic aberrations regardless of sex and gender.

### Materials

All compounds were purchased from MedChemExpress (Inobrodib) or TOCRIS (A-485). CP-C27 was prepared using a method similar to that used for compound 21 (19, 21). The ON-TARGETplus SMARTPool siRNAs specific for target genes *CREBBP* (L-003477-00), *EP300* (L-003486-00), *KREMEN2* (L-003847-00), and *KREMEN1* (L-003846-00) were purchased from Dharmacon.

### Cell viability assay

To measure cell viability after siRNA transfection, cells were trypsinized, counted, and reseeded in 24-well plates at a density of 1 to 2 × 10^5^ cells per well. Next, the cells were transfected with siRNAs (50 nmol/L) using Lipofectamine RNAiMAX (Thermo Fisher Scientific; 13778150). After 48 hours, the cells were trypsinized again, reseeded in 24-well plates, and transfected repeatedly with siRNAs (50 nmol/L) using Lipofectamine RNAiMAX. The cells were trypsinized after 48 hours, counted, and reseeded in 96-well plates at a density of 500 cells per well. After 7 days, cell viability was examined by measuring ATP levels in the CellTiter-Glo Luminescent Cell Viability Assay (Promega, G7571). Cell viability was measured after treatment with an inhibitor, and the cells were trypsinized, counted, and reseeded in 96-well plates at a density of 500 cells per well. After 24 hours, the cells were treated with the inhibitors at the indicated concentrations. After 6 days, cell viability was measured in the CellTiter-Glo Luminescent Cell Viability Assay (Promega, G7571). Cell viability was measured after treatment with an inhibitor and transfection with a siRNA, and the cells were transfected for 48 hours with the indicated siRNAs. The cells were then transfected with the siRNAs for an additional 24 hours. The cells were then reseeded again and treated with CP-C27. After 4 days, cell viability was measured in the CellTiter-Glo Luminescent Cell Viability Assay (Promega, G7571). Luminescence was measured using a Nivo plate reader (RRID: SCR_025763; PerkinElmer). IC_50_ values were calculated using GraphPad Prism 8 (RRID: SCR_002798).

### Quantitation of mRNA

A measure of 2 × 10^4^ cells were plated into 96-well plates to measure basal mRNA levels and incubated for 24 hours. For drug treatment, 2 × 10^4^ cells were plated in 96-well plates and incubated for 24 hours. The medium was then replaced with medium containing (or not) A-485 or CP-C27 and incubated for 24 hours. To establish siRNA-transfected cells, 2 × 10^4^ cells were plated into 96-well plates, transfected with siRNAs (50 nmol/L) using Lipofectamine RNAiMAX (Thermo Fisher Scientific; 13778150), and incubated for 48 or 96 hours. First, mRNA was extracted from all cell lines, and cDNA was synthesized using SuperPrep II Cell Lysis & RT Kit for qPCR (TOYOBO; SCQ-401). Aliquots of cDNA were subjected to qPCR using THUNDERBIRD Probe qPCR Mix (TOYOBO; QPS101) and TaqMan Gene Expression Assays (Thermo Fisher Scientific). Before extraction of mRNA, tumor xenograft samples were weighed and washed with PBS. Samples were cut into 3-mm squares (30 mg) and milled in liquid nitrogen before extraction of mRNA using the Animal Tissues protocol from RNeasy Mini Kit (QIAGEN; 74104) and a QIAshredder (QIAGEN; 79654); the final elution volume was 50 μL. Next, cDNA was synthesized using PrimeScript RT Master Mix (Perfect Real Time; Takara; RR036A). Primer/probe sets specific for the following genes were used for TaqMan Gene Expression Assays: *CASP6* (Hs00154250_m1), *CREBBP* (Hs00932878_m1), *EP300* (Hs00914212_m1), *KREMEN1* (Hs00230750_m1), *KREMEN2* (Hs00225867_m1), and *GAPDH* (Hs99999905_m1; Thermo Fisher Scientific). PCR was performed in ABI StepOnePlus Real-Time PCR System (RRID: SCR_015805; Applied Biosystems) under the following conditions: denaturation at 95°C for 15 seconds, followed by annealing and extension at 60°C for 30 seconds (40 cycles). For each sample, the mRNA level of target genes was normalized to that of *GAPDH*. The target/GAPDH ratios were then normalized against those in control samples using the 2−ΔΔCt method.

### Western blot analysis

A measure of 5 × 10^5^ cells were harvested, washed with PBS, and lysed at 95°C for 5 minutes with 150 μL of 1× SDS sample buffer to extract proteins. Chromatin was sonicated on ice (20 cycles of 15-second pulses; high setting; 15 seconds between pulses) using a Bioruptor (M&S Instruments). Samples of tumor xenografts were lysed using Precellys Evolution (M&S Instruments) with 500 μL of RIPA buffer (CST, 9806) supplemented with Halt proteinase and phosphatase inhibitor cocktail (Thermo Fisher Scientific, 78440). The lysates were incubated for 5 minutes on ice, followed by centrifugation for 10 minutes at 12,000 rpm. The supernatants were mixed with 250 μL of 3× SDS sample buffer and boiled at 95°C for 5 minutes. The cell lysates were quantified using Pierce 660 nm Protein Assay Reagent (Thermo Fisher Scientific, 22660) and Ionic Detergent Compatibility Reagent for Pierce 660 nm Protein Assay Reagent (Thermo Fisher Scientific, 22663). Next, 15 μg of protein was analyzed by immunoblotting. Proteins were separated by SDS-PAGE, transferred to polyvinylidene difluoride (PVDF) membranes, and immunoblotted with the indicated antibodies. β-actin was used as a loading control. Briefly, the membranes were blocked for 1 hour at 25°C with PVDF Blocking Reagent for Can Get Signal (TOYOBO, NYPBR01) and then probed for 1 hour at 25°C with Can Get Signal Solution 1 (TOYOBO, NKB-201) containing primary antibodies. After washing with TBS containing 0.1% Tween 20, the membranes were incubated for 30 minutes at 25°C with TBS containing 0.1% Tween 20, 1% BSA, and horseradish peroxidase–conjugated anti-mouse (CST, 7076) or anti-rabbit (CST, 7074) secondary antibodies before visualization using Western Lightning ECL Pro (Perkin Elmer, NEL120001EA) or the ECL Prime Western Blotting System (Cytiva, RPN2232). Chemiluminescence signals were measured using a FUSION Solo S chemiluminescence imaging system (M&S Instruments) or Amersham Imager 600 (RRID: SCR_021853; Cytiva). Antibodies specific for the following proteins were used for immunoblotting: SMARCA4 (1:1,000, CST, 49360, RRID: AB_2728743), SMARCA2 (1:1,000, CST, 11966, RRID: AB_2797783), SMARCB1 (1:1,000, CST, 91735, RRID: AB_2800172), ARID1A (1:1,000, CST, 12354, RRID: AB_2637010), PBRM1 (1:1,000, CST, 89123, RRID: AB_2936366), SS18 (1:1,000, CST, 21792, RRID: AB_2728667), SS18–SSX (1:1,000, CST, 70929, RRID: AB_2799794), CBP (1:1,000, CST, 7425, RRID: AB_10949975), p300 (1:1,000, CST, 54062, RRID: AB_2799450), H3K27ac (1:1,000, CST, 8173, RRID: AB_10949503), and β-actin (1:2,000, CST, 5125, RRID: AB_1903890).

### Gene expression analysis from DepMap

**Figure 1:**
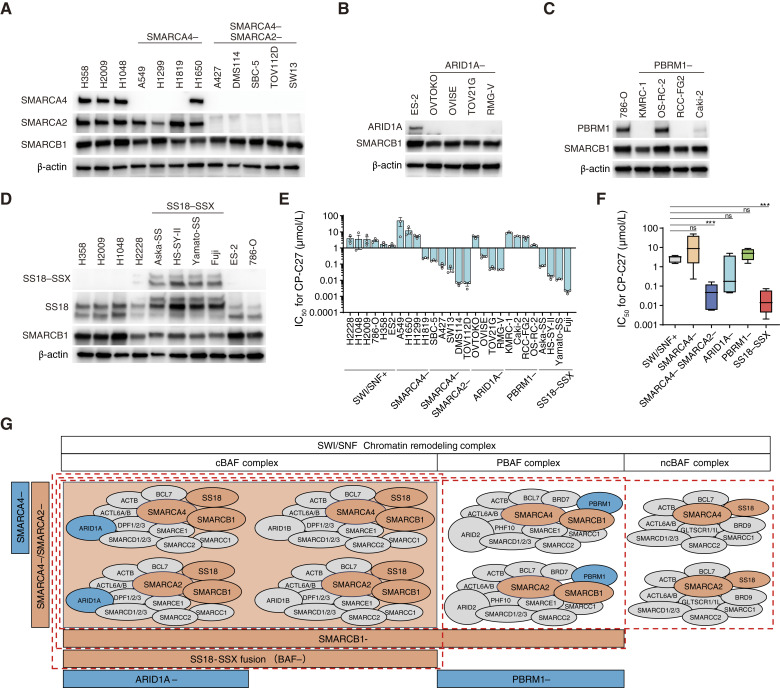
CBP and p300 dual inhibitors selectively sensitize cancer cells deficient in a subunit of the cBAF complex. **A,** Immunoblot analysis of SMARCA4, SMARCA2, SMARCB1, and β-actin expression in SWI/SNF-proficient, SMARCA4-deficient, and SMARCA4/SMARCA2-deficient cell lines. **B,** Immunoblot analysis of ARID1A, SMARCB1, and β-actin expression in SWI/SNF-proficient and ARID1A-deficient cell lines. **C,** Immunoblot analysis of PBRM1, SMARCB1, and β-actin expression in SWI/SNF-proficient and PBRM1-deficient cell lines. **D,** Immunoblot analysis of SMARCA4, SMARCA2, SMARCB1, and β-actin expression in SWI/SNF-proficient and SS18–SSX fusion cell lines. **E** and **F,** IC_50_ values for CBP/p300 inhibitor CP-C27 in SWI/SNF-proficient (H2228, H1048, H2009, 786-O, H358, and ES-2), SMARCA4-deficient (A549, H1650, H1299, and H1819), SMARCA4/SMARCA2-deficient (SBC-5, A427, SW-13, DMS114, and TOV112D), ARID1A-deficient (OVTOKO, OVISE, TOV-21G, and RMG-V), PBRM1-deficient (KMRC-1, Caki-2, RCC-FG2, and OS-RC-2), and SS18–SSX fusion (Aska-SS, HS-SY-II, Yamato-SS, and Fuji) cell lines (**E**) and cell line groups (**F**). Cells were treated with inhibitors for 6 days, and IC_50_ values were calculated based on cell viability. Data are presented as the mean ± SEM; *n* = 3 for the independent experiments in **E**. In **F**, the number of cell line groups was as follows: SWI/SNF-proficient (*n* = 6), SMARCA4-deficient (*n* = 4), SMARCA4/SMARCA2-deficient (*n* = 5), ARID1A-deficient (*n* = 4), PBRM1-deficient (*n* = 4), and SS18–SSX fusion (*n* = 4). **G,** Schematic diagram of the SWI/SNF subcomplexes showing sensitivity to CBP/p300 inhibitors. SWI/SNF chromatin remodeling complexes can be classified broadly into three subcomplexes: cBAF, PBAF, and ncBAF. When focused on SMARCB1, SMARCA4, SMARCA2, SS18, ARID1A, and PBRM1, SMARCB1 is included in four subcomplexes of cBAF and two subcomplexes of PBAF; both SMARCA4 and SMARCA2 are included in all subcomplexes; SS18 is included in four subcomplexes of cBAF and two subcomplexes of ncBAF; and SMARCB1, SMARCA4, SMARCA2, and SS18 are common subunits of cBAF. Because SMARCB1-deficient, SMARCA4/SMARCA2-deficient, and SS18–SSX fusion cell lines are highly sensitive to CBP/p300 dual inhibitors, it is conceivable that cancers harboring abnormal constituent genes within the four subcomplexes of the cBAF complex may be sensitive to CBP/p300 dual inhibitors. For all experiments, *P* values were determined by an unpaired two-tailed Student *t* test. *, *P* < 0.05; **, *P* < 0.01; ***, *P* < 0.001.

Mutation, copy number (CN), and gene expression datasets were obtained from the Cancer Cell Line Encyclopedia (CCLE) database and downloaded from the DepMap website [RRID: SCR_017655; data version 23Q2, (http://www.depmap.org/)]. These downloaded data were analyzed as follows. To examine the expression of the *KREMEN2* gene among cell lines with different genetic abnormalities, mutation and CN data were combined to infer the genotype; this was done because tumor suppressor genes, such as *SMARCB1*, *ARID1A*, *PBRM1*, and *SMARCA4*, can be lost through a combination of mutations and CN deletions. Specifically, the following methods were used to determine the genotype of *SMARCB1*, *ARID1A*, *PBRM1*, *SMARCA4*, *SMARCA2*, or *SS18–SSX* in terms of the number of altered allele(s) with mutations and/or CN deletions. First, values (0, 1, or 2) stored in the table of damaging mutations from DepMap were used to obtain the number of mutated alleles. The number of deleted alleles was defined as follows: when the measured CN was <0.5, the gene was defined as being homozygously deleted (deleted alleles 2); when the measured CN was <1.5 but ≥0.5, the gene was defined as being heterozygously deleted (deleted alleles 1). The number of mutated and deleted alleles was then summed up to calculate the number of altered alleles. For a fraction of cell lines in which the calculated altered allele numbers exceeded 2, the numbers were set to 2. Cell lines with altered alleles were considered SMARCB1-, ARID1A-, PBRM1-, SMARCA4-, SMARCA2-, and SMARCA4/SMARCA2-deficient or harboring the SS18–SSX fusion. Because this assignation correctly determined SMARCB1 deficiency in seven of nine reported SMARCB1-deficient cell lines (22), it was applied to all cell lines in the CCLE. Although SMARCA4/SMARCA2-deficient cell lines often do not have SMARCA2 mutations, the expression of SMARCA2 is silenced. Thus, based on genetic abnormality data alone, SMARCA4/SMARCA2-deficient cell lines cannot be often detected. Therefore, A427, DMS114, SW-13, TOV112D (Fig. 1A), COV434 (23), and NCIH23 (24) cell lines deficient in SMARCA4 and SMARCA2 proteins were classified as SMARCA4/SMARCA2-deficient cell lines. Finally, the expression of the *KREMEN2* gene in deficient/fusion and nondeficient cell lines was compared.

### Annexin V/propidium iodide staining assay

Annexin V–FITC/PI Apoptosis Detection Kit (Roche, 11858777001) was used to detect apoptotic cells. For drug-treated cells, 1 × 10^5^ cells were plated into 24-well plates, incubated for 24 hours, and treated without or with A-485 for 6 days. The cell pellets were washed with PBS, suspended in 1× binding buffer, and then incubated for 10 minutes at 25°C in the dark with annexin V–FITC and propidium iodide. Fluorescence was analyzed on a Guava easyCyte HT system (RRID: SCR_025377; Millipore). The gating of live cells was based on side scatter (SCC) and forward scatter (FSC) area parameters. annexin V–FITC–positive fractions were detected by assessing the percentage of cells for which the signal intensity was higher than that of cell fractions abundant in nontreated samples. The percentage of annexin –-positive cells was calculated using GuavaSoft software (v. 2.7).

### Processing of next-generation sequencing data

Raw sequencing data from chromatin immunoprecipitation sequencing (ChIP-seq) and Assay for Transposase-Accessible Chromatin using sequencing (ATAC-seq) were trimmed using fastp version 0.12.4 (RRID: SCR_016962; ref. 25) and mapped to the human reference genome (hg38) using Bowtie2 version 2.4.5 (RRID: SCR_016368), with parameters -k 1 –no-mixed –no-discordant -X 2000 (26). Before all downstream analyses, duplicate reads were removed using the MarkDuplicates command in Picard version 2.26.11 (RRID: SCR_006525; http://broadinstitute.github.io/picard). CPM values in the genome tracks were calculated from the ChIP-seq data by subtracting the input tracks as the background value for each cell. Raw sequencing data derived from RNA sequencing (RNA-seq) were trimmed using fastp version 0.12.4 (25) and mapped to the hg38 genome using HISAT2 version 2.2.1 (RRID: SCR_015530; ref. 27). BigWig files for ChIP-seq data were generated using the bamCompare command in deepTools version 3.5.1 (RRID: SCR_016366), with parameters –operation subtract –normalizeUsing CPM –scaleFactorsMethod None –binSize 10 –smoothLength 30 (28). BigWig files for the ATAC-seq and RNA-seq data were generated using the bamCoverage command from deepTools version 3.5.1 (RRID: SCR_016366), with parameters –normalizeUsing CPM –binSize 10 –smoothLength 30 (28). BigWig files were visualized by Integrative Genomics Viewer version 2.13.2 (RRID: SCR_011793; ref. 29).

### Mouse xenograft model

Cells were counted and resuspended in a 1:1 mixture of PBS/Matrigel (Corning, 354234; 100 μL:100 μL, or 25 μL:25 μL) on ice. After that, cells [DMS114 (1 × 10^6^ cells/mouse), Fuji (5 × 10^6^ cells/mouse), A427 (3 × 10^6^ cells/mouse), or TOV112D (1 × 10^6^ cells/mouse)] were injected subcutaneously into the flank of 5 to 6-week-old female BALB/c-nu/nu mice or SCID mice (CLEA or Jackson Laboratory). The mice were randomized into two groups when the tumors were palpable (about 14–21 days after implantation). Mice in the drug treatment group were injected orally with either vehicle [PBS or 0.5 w/v% methyl cellulose 400 solution (FUJIFILM Wako, 133-17815)] or CP-C27 (30–50 mg/kg) once daily or twice daily for 14 to 28 days. Tumor growth was measured every few days using calipers. The volume of implanted tumors was calculated using the formula *V* = *L* × *W*^2^/2, in which *V* is the volume (mm^3^), *L* is the largest diameter (mm), and *W* is the smallest diameter (mm). At the end of the experiment, mice were sacrificed in accordance with standard protocols. For pharmacokinetics analysis, blood and tumor tissues were harvested 2, 4, and 8 hours after the final administration. Plasma was prepared from blood by centrifugation at 3,000 × *g* for 10 minutes at 4°C. The plasma samples were stored at −80°C until required for measurements. The tumor homogenate was prepared by adding methanol (a volume of 4 times the tumor weight) to the collected tumor, followed by crushing at 6,000 × *g* for 20 seconds with a bead homogenizer (TOMY) under cooling. The concentration of the plasma samples and the tumor homogenates was measured by LC/MS (SCIEX).

### IHC

Xenografts were fixed immediately in a 10% neutral buffered formalin solution. After 24 hours, the xenografts were set in tissue-processing cassettes measuring 3.5 × 2.5 × 0.4 cm. The specimens were dehydrated by passage through a series of ethanol solutions, beginning with 70% ethanol and finishing with 100% ethanol. Next, the ethanol in the tissue was replaced by xylene, miscible with paraffin. Finally, the tissue specimens were infiltrated and embedded in paraffin. The steps from dehydration to paraffin infiltration were fully automated and performed in a Tissue-Tek VIP 6 AI apparatus (Sakura Finetek). Then, the paraffin blocks were sectioned (4 μm thick) before hematoxylin and eosin and IHC staining. Tissue sections were stained using antibodies specific for cleaved caspase-3 (Asp175; clone 5A1E, #9664, diluted 1:200, antigen retrieval in citrate buffer; Cell Signaling Technology) and cleaved PARP (Asp214; clone D64E10, #5625, diluted 1:100, antigen retrieval in citrate buffer; Cell Signaling Technology). All IHC staining was performed using a Dako autostainer Link48 (Agilent Technologies). Cleaved caspase-3 was evaluated only in nonnecrotic areas, and the percentage of cytoplasm-positive tumor cells within the total tumor cell population was calculated. The number of cleaved PARP–positive cells within the total number of viable cells per slide was evaluated, and the mean number of positive tumor cells in three high-power fields (×400) within a hotspot of the tumor tissue was reported.

### Statistical analysis

Statistical analyses were performed using Microsoft Excel (RRID: SCR_016137) or GraphPad Prism 8 (RRID: SCR_002798). As the figure legends indicate, data are expressed as the mean ± SD or mean ± SEM. The sample size (*n*) is indicated in the figure legends and represents the number of biological or technical replicates. Statistical significance was evaluated using a two-tailed Student *t* test. Statistically significant differences are indicated by asterisks as follows: *, *P* < 0.05; **, *P* < 0.01; ***, *P* < 0.001.

### Data availability

The ChIP-seq, ATAC-seq, and RNA-seq datasets for the BIN-67 and Aska-SS cell lines were obtained from publicly available NCBI Gene Expression Omnibus (GEO) datasets (RRID: SCR_005012; GSE117735, GSE108028; refs. 30, 31). These downloaded data were analyzed as described in the next-generation sequencing data processing section. Mutation, CN, and gene expression datasets were obtained from the CCLE database and downloaded from the DepMap website [RRID: SCR_017655; data version 23Q2, (http://www.depmap.org/)]. The data generated in this study are available upon request from the corresponding author.

## Results

### CBP and p300 dual inhibitors selectively sensitize cancer cells deficient in a subunit of the cBAF complex

Recently, we showed that treatment with simultaneous inhibitors of paralog pair CBP/p300 induces synthetic lethality in SMARCB1-deficient cancers (19). *SMARCB1* is a constituent gene of SWI/SNF, although other constituent genes in various cancers also harbor genetic abnormalities. Therefore, we examined the potential for expansion/adaptation of CBP/p300 dual inhibitors to cancers carrying abnormal SWI/SNF constituent genes. First, we constructed a cell line panel comprising a SWI/SNF-proficient type (SWI/SNF+), a SMARCA4 single-deficient type (SMARCA4−), a SMARCA4 and SMARCA2 double-deficient type (SMARCA4−SMARCA2−), an ARID1A-deficient type (ARID1A−), a PBRM1-deficient type (PBRM1−), and an SS18–SSX fusion type (SS18–SSX; Fig. 1A–D; Supplementary Fig. S1A). These cell lines examined sensitivity to the CBP/p300 dual inhibitor CP-C27. The IC_50_ values for CP-C27 in the SMARCA4/SMARCA2-deficient (SMARCA4−SMARCA2−) cell line group (SBC-5, A427, SW-13, DMS114, and TOV112D) and the SS18–SSX fusion cell line group (Aska-SS, HS-SY-II, Yamato-SS, and Fuji) were lower than those in the SWI/SNF-proficient cell line group (H2228, H1048, H2009, 786-O, H358, and ES2; Fig. 1E and F). By contrast, the IC_50_ values for CP-C27 in the SMARCA4-deficient cell line group [A549, H1299, H1819, and H1650 (point mutant type: SMARCA4N1164Y)], the ARID1A-deficient cell line group (OVTOKO, OVISE, TOV-21G, and RMG-V), and the PBRM1-deficient cell line group [KMRC-1, Caki-2, RCC-FG2, and OS-RC-2 (point mutant type: PBRM1I233T)] were comparable with those in the SWI/SNF-proficient cell line group (Fig. 1E and F). This data indicates that SMARCA4/SMARCA2-deficient and SS18–SSX fusion cell lines are highly sensitive to CP-C27. This result is similar to that reported for SMARCB1-deficient cell lines (19). In addition, the SMARCA4/SMARCA2-deficient cell line group and the SS18–SSX fusion cell line group were more sensitive to A-485 and Inobrodib, another CBP/p300 dual inhibitor, than the SWI/SNF-proficient cell line group (Supplementary Fig. S1B and S1C). These results suggest that CBP/p300 dual inhibitors are highly effective against SMARCB1-deficient cell lines but also against SMARCA4/SMARCA2-deficient cell lines and SS18–SSX fusion cell lines.

SWI/SNF chromatin remodeling complexes can be classified broadly into three subcomplexes: cBAF, PBAF, and ncBAF (Fig. 1G; ref. 6). If we focus on SMARCB1, SMARCA4, SMARCA2, SS18, ARID1A, and PBRM1, we find that SMARCB1 is included in four subcomplexes of cBAF and two subcomplexes of PBAF, that SMARCA4 and SMARCA2 together are included in all subcomplexes; and that SS18 is included in four subcomplexes of cBAF and two subcomplexes of ncBAF. In synovial sarcoma cell lines harboring an SS18–SSX fusion, degradation of the BAF complex is promoted, although the stability of the ncBAF complex is not affected; this suggests that the function of the BAF complex is suppressed (32). SMARCB1, SMARCA4, SMARCA2, and SS18 are common subunits of cBAF subcomplexes. Because SMARCB1-deficient, SMARCA4/SMARCA2-deficient, and SS18–SSX fusion cell lines are highly sensitive to CBP/p300 dual inhibitors, it is conceivable that cancers harboring abnormal constituent genes in all of the four subcomplexes of the cBAF complex could be highly sensitive to CBP/p300 dual inhibitors (Fig. 1G).

### Simultaneous inhibition of CBP and p300 causes synthetic lethality in SMARCA4/SMARCA2-deficient and SS18–SSX fusion cells

**Figure 2:**
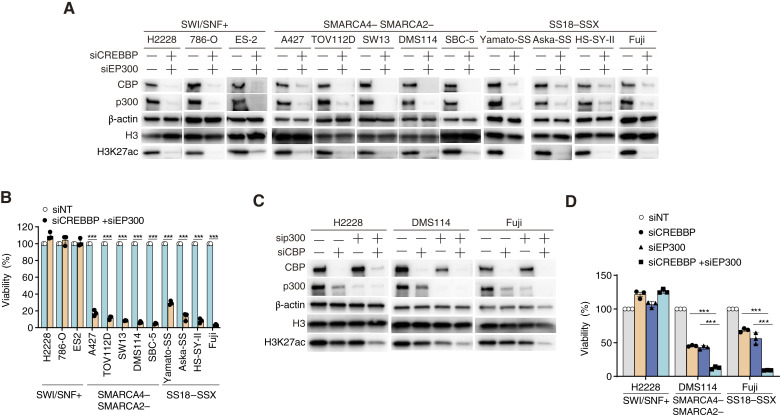
Simultaneous inhibition of CBP/p300 causes synthetic lethality in SMARCA4/SMARCA2-deficient and SS18–SSX fusion cells. **A,** Immunoblot analysis of CBP, p300, H3K27ac, H3, and β-actin expression in SWI/SNF-proficient, SMARCA4/SMARCA2-deficient, and SS18–SSX fusion cell lines transfected for 48 hours with the indicated siRNAs. **B,** Viability of SWI/SNF-proficient, SMARCA4/SMARCA2-deficient, and SS18–SSX fusion cell lines transfected for 48 hours with the indicated siRNAs. The cells were then reseeded and transfected repeatedly with the indicated siRNAs for 48 hours. The cells were then reseeded again and incubated for 7 days. Data are presented as the mean ± SEM; *n* = 3 independent experiments. **C,** Immunoblot analysis of CBP, p300, H3K27ac, H3, and β-actin expression in SWI/SNF-proficient H2228, SMARCA4/SMARCA2-deficient DMS114, and SS18–SSX fusion Fuji cell lines transfected for 48 hours with the indicated siRNAs. **D,** Viability of SWI/SNF-proficient H2228, SMARCA4/SMARCA2-deficient DMS114, and SS18–SSX fusion Fuji cell lines transfected for 48 hours with the indicated siRNAs. The cells were then reseeded and transfected repeatedly with the indicated siRNAs for 48 hours. The cells were then reseeded again and incubated for 7 days. Data are presented as the mean ± SEM; *n* = 3 independent experiments. For all experiments, *P* values were determined by an unpaired two-tailed Student *t* test. *, *P* < 0.05; **, *P* < 0.01; ***, *P* < 0.001.

Next, we examined the effects of simultaneously suppressing *CREBBP*/*EP300* at the gene level on cell proliferation. Simultaneous depletion of the paralog pair *CREBBP*/*EP300* decreased the viability of five SMARCA4/SMARCA2-deficient cell lines (A427, TOV112D, SW-13, DMS114, and SBC-5) and four SS18–SSX fusion cell lines (Yamato-SS, Aska-SS, HS-SY-II, and Fuji) but not that of three SWI/SNF-proficient cell lines (H2228, 786-O, and ES2), i.e., synthetic lethality (Fig. 2A and B).

Next, we asked whether dual suppression of *CREBBP* and *EP300*, but not that of either alone, causes synthetic lethality. Depleting either *CREBBP* or *EP300* (Fig. 2C; Supplementary Fig. S2A and S2B) partially suppressed the growth of SMARCA4/SMARCA2-deficient DMS114 cells and SS18–SSX fusion Fuji cells (Fig. 2D). Moreover, simultaneous depletion of both paralogs in SMARCA4/SMARCA2-deficient cells and SS18–SSX fusion cells (Fig. 2C; Supplementary Fig. S2A and S2B) led to significantly greater growth suppression than depletion of either paralog alone (Fig. 2D); however, single or dual depletion of *CREBBP* and *EP300* (Fig. 2C; Supplementary Fig. S2A and S2B) did not affect the growth of SWI/SNF-proficient H2228 cells (Fig. 2D). Thus, simultaneous suppression of *CREBBP* and *EP300*, but not that of either alone, causes synthetic lethality in SMARCA4/SMARCA2-deficient and SS18–SSX fusion cancers.

### SMARCA4/SMARCA2 deficiency and SS18–SSX fusion lead to upregulation of the *KREMEN2* gene

**Figure 3:**
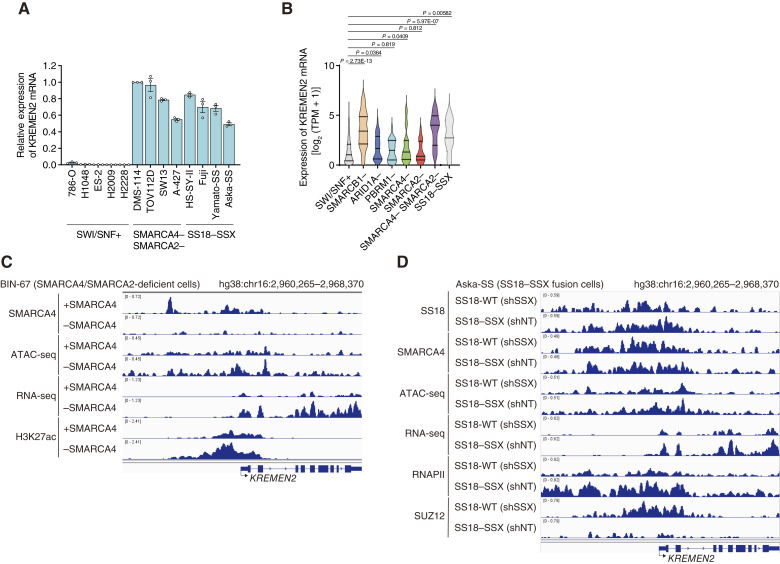
SMARCA4/SMARCA2 deficiency and SS18–SSX fusion lead to upregulation of *KREMEN2* gene expression. **A,** Expression of *KREMEN2* mRNA in SWI/SNF-proficient, SMARCA4/SMARCA2-deficient, and SS18–SSX fusion cell lines (relative to that in DMS114 cells). Data are presented as the mean ± SEM; *n* = 3 independent experiments. **B,** Violin plots showing clustered expression of *KREMEN2* mRNA in SWI/SNF-WT, SMARCB1-deficient, ARID1A-deficient, PBRM1-deficient, SMARCA4-deficient, SMARCA4/SMARCA2-deficient, and SS18–SSX fusion cell lines based on an analysis of mutations, gene CN, and gene expression data from DepMap (data version 23Q2). SWI/SNF-proficient (*n* = 1,238), *SMARCB1*-deficient (*n* = 19), ARID1A-deficient (*n* = 52), PBRM1-deficient (*n* = 23), SMARCA4-deficient (*n* = 34), SMARCA2-deficient (*n* = 27), SMARCA4/SMARCA2-deficient (*n* = 6), and SS18–SSX (*n* = 5) cell lines were analyzed, and data are presented as the mean ± SEM. *n* indicates the number of independent samples. **C,** Localization of signals generated by SMARCA4 and H3K27ac ChIP-seq, ATAC-seq, and RNA-seq around the *KREMEN2* locus in SMARCA4/SMARCA2-deficient BIN-67 cells transduced with an SMARCA4 expression vector (+SMARCA4) and in BIN-67 cells without the SMARCA4 expression vector (−SMARCA4). **D,** Localization of signals generated by SS18, SMARCA4, RNAPII, and SUZ12 ChIP-seq, ATAC-seq, and RNA-seq around the *KREMEN2* locus in SS18–SSX-fusion Aska-SS cells depleted of SS18–SSX by *SSX*-targeting shRNA (shSSX; SS18-WT) and in SS18–SSX fusion Aska-SS cells not depleted of SS18–SSX [i.e., transduced with NT shRNA (shNT; SS18–SSX)].

Through our previous investigation of downstream factors influencing synthetic lethality from simultaneous inhibition of CBP/p300 in SMARCB1-deficient cells, we discovered that the *KREMEN2* gene was uniquely upregulated in SMARCB1-deficient cells (19). KREMEN2 is a single-pass transmembrane protein that plays a role in suppressing the apoptosis pathway via interaction with KREMEN1 (19, 33, 34); therefore, we examined the expression of *KREMEN2* mRNA in SMARCA4/SMARCA2-deficient and SS18–SSX fusion cell lines. Expression of *KREMEN2* mRNA in SMARCA4/SMARCA2-deficient and SS18–SSX fusion cell lines was higher than that in SWI/SNF-proficient cell lines (Fig. 3A). Expression of *KREMEN2* mRNA in ARID1A-deficient, PBRM1-deficient, and SMARCA4-deficient cell lines was comparable with that in SWI/SNF-proficient cell lines but lower than that in SMARCA4/SMARCA2-deficient and/or SS18–SSX fusion cell lines (Supplementary Fig. S3A and S3B). These results indicate that SMARCA4/SMARCA2 and SS18 are required for transcriptional repression of *KREMEN2* and that SMARCA4/SMARCA2 deficiency and SS18–SSX fusion increases expression of *KREMEN2* mRNA due to derepression.

To validate expression levels of the *KREMEN2* gene among cell lines with different genetic abnormalities, we used mutation, CN, and gene expression data from the CCLE database in DepMap (data version 23Q2). Expression of *KREMEN2* mRNA was compared with gene expression data derived from SWI/SNF-proficient, SMARCB1-deficient, ARID1A-deficient, PBRM1-deficient, SMARCA4-deficient, SMARCA2-deficient, SMARCA4/SMARCA2-deficient, and SS18–SSX fusion cell lines. The results confirmed that expression of *KREMEN2* in SMARCA4/SMARCA2-deficient and SS18–SSX fusion cell lines, as well as in SMARCB1-deficient cell lines, was significantly higher than that in SWI/SNF-proficient cell lines (Fig. 3B).

To further investigate transcriptional regulation at the *KREMEN2* gene locus, we used published ChIP-seq data (GSE117735; ref. 30) derived from the SMARCA4 isogenic model of SMARCA4/SMARCA2-deficient BIN-67 to characterize the chromatin state at the locus (Fig. 3C). SMARCA4 was localized at the regions upstream of the *KREMEN2* locus (Fig. 3C). In SMARCA4-rescued BIN-67 cells (+SMARCA4), localization of ATAC-seq signals near the transcription start site (TSS) of the *KREMEN2* locus was reduced, and RNA-seq signals were weaker than those for SMARCA4-deficient BIN-67 cells (−SMARCA4; Fig. 3C). In SMARCA4-rescued BIN-67 cells (+SMARCA4), RNA-seq signals were weaker than those for SMARCA4-deficient BIN-67 cells (−SMARCA4; Fig. 3C). In SMARCA4-rescued BIN-67 cells (+SMARCA4), localization of histone H3K27ac, a histone acetylated by CBP and p300, and a marker of transcriptionally activated regions near the TSS of the *KREMEN2* locus was decreased (Fig. 3C). These results suggest that SMARCA4 represses transcription of *KREMEN2* by localizing to the transcriptional region of the *KREMEN2* locus and that transcription of *KREMEN2* is promoted when SMARCA4 is lost.

Furthermore, we used published ChIP-seq data (GSE108028; ref. 31) derived from SS18–SSX fusion Asaka-SS cells to characterize the chromatin state at the locus (Fig. 3D). In the Aska-SS cell line, knockdown of SS18–SSX1 by fusion-specific shRNA (sh*SSX*) rescued expression of the SS18 wild-type (WT; ref. 31), indicating that SS18-WT is expressed in Aska-SS sh*SSX* cells (sh*SSX*) and that the SS18–SSX fusion protein is expressed in Aska-SS sh nontargeting (NT) cells (shNT). To identify the consequences of SS18–SSX protein expression, we analyzed ChIP-seq data obtained after SS18–SSX fusion–specific (sh*SSX*) knockdown. We compared them with shNT data from the Aska-SS cell line. The SS18-WT protein in Aska-SS sh*SSX* cells (harboring SS18-WT) localized upstream of the TSS of the *KREMEN2* locus (Fig. 3D). Unexpectedly, the SS18–SSX fusion protein in Aska-SS shNT cells (harboring SS18–SSX) also localized at similar regions (Fig. 3D). SMARCA4 also localized to the SS18-localized regions at the *KREMEN2* locus (Fig. 3D); however, ATAC-seq signals near the TSS of the *KREMEN2* locus in Aska-SS sh*SSX* (with SS18-WT) cells were more attenuated than that in Aska-SS shNT (harboring SS18–SSX) cells (Fig. 3D). RNA-seq read counts in Aska-SS sh*SSX* (harboring SS18-WT) cells were lower than those in Aska-SS shNT (harboring SS18–SSX) cells (Fig. 3D). In addition, localization of RNAPII near the TSS locus at the *KREMEN2* locus in Aska-SS sh*SSX* (harboring SS18-WT) cells was lower than that in Aska-SS shNT (harboring SS18–SSX) cells (Fig. 3D). By contrast, localization of the transcriptional repressor SUZ12, a component of the PRC2 transcriptional repressor complex, near the TSS of the *KREMEN2* locus was higher in Aska-SS shSSX (harboring SS18-WT) cells than in Aska-SS shNT (harboring SS18–SSX) cells (Fig. 3D). These results suggest that transcription of *KREMEN2* is repressed in SS18-WT cells but promoted in SS18–SSX cells. When the cBAF and/or ncBAF complex containing SS18-WT is localized to the transcriptional regulatory region of the *KREMEN2* locus, it represses transcription of *KREMEN2*. By contrast, in SS18–SSX cells, the ncBAF complex, including SS18–SSX, localized to the transcriptional regulatory region of the *KREMEN2* locus because the SS18-WT protein is degraded and the cBAF complex is disintegrated in SS18–SSX cells (31). These data indicate that *KREMEN2* expression is upregulated through derepression caused by SMARCA4/SMARCA2 deficiency and SS18–SSX fusion.

### Simultaneous inhibition of CBP/p300 in SMARCA4/SMARCA2-deficient and SS18–SSX fusion cells induces apoptosis by downregulating *KREMEN2*

**Figure 4:**
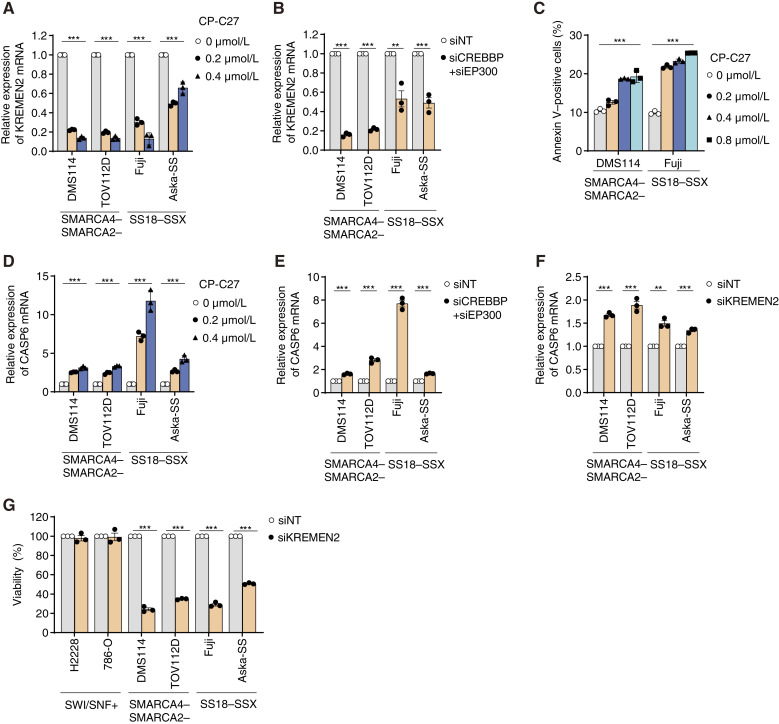
Simultaneous inhibition of CBP/p300 in SMARCA4/SMARCA2-deficient and SS18–SSX fusion cells induces apoptosis by downregulating *KREMEN2*. **A,** Expression of *KREMEN2* mRNA (relative to that in nontreated cells) in SMARCA4/SMARCA2-deficient and SS18–SSX fusion cells treated for 24 hours with the indicated concentrations of CP-C27. Data are presented as the mean ± SD; *n* = 3 independent experiments. **B,** Expression of *KREMEN2* mRNA (relative to that in siNT-transfected cells) in SMARCA4/SMARCA2-deficient and SS18–SSX fusion cells transfected for 48 hours with the indicated siRNAs. Data are presented as the mean ± SD; *n* = 3 independent experiments. **C,** Percentage of annexin V–positive cells within the SMARCA4/SMARCA2-deficient and SS18–SSX fusion cell populations treated for 6 days with the indicated concentrations of CP-C27. Data are presented as the mean ± SD; *n* = 3 independent experiments. **D,** Expression of *CASP6* mRNA (relative to that in nontreated cells) in SMARCA4/SMARCA2-deficient and SS18–SSX fusion cells treated for 24 hours with the indicated concentrations of CP-C27. Data are presented as the mean ± SD; *n* = 3 independent experiments. **E,** Expression of *CASP6* mRNA (relative to that in siNT-transfected cells) in SMARCA4/SMARCA2-deficient and SS18–SSX fusion cells transfected for 48 hours with the indicated siRNAs. Data are presented as the mean ± SD; *n* = 3 independent experiments. **F,** Expression of *CASP6* mRNA (relative to that in siNT-transfected cells) in SMARCA4/SMARCA2-deficient and SS18–SSX fusion cells transfected for 48 hours with the indicated siRNAs. Data are presented as the mean ± SD; *n* = 3 independent experiments. **G,** Viability of SMARCA4/SMARCA2-deficient and SS18–SSX fusion cells transfected with the indicated siRNAs. Cells were transfected for 48 hours with the indicated siRNAs. Cells were then reseeded and transfected with the indicated siRNAs for 48 hours. The cells were then reseeded again and incubated for 7 days. Data are presented as the mean ± SEM; *n* = 3 independent experiments. For all experiments, *P* values were determined by an unpaired two-tailed Student *t* test. *, *P* < 0.05; **, *P* < 0.01; ***, *P* < 0.001.

Next, we investigated whether upregulation of *KREMEN2* expression via derepression caused by SMARCA4/SMARCA2 deficiency and SS18–SSX fusion depends on CBP/p300. Treatment of cells with CBP/p300 dual inhibitors CP-C27 and A-485 downregulated expression of *KREMEN2* mRNA in SMARCA4/SMARCA2-deficient cells (DMS114 and TOV112D) and SS18–SSX fusion cells (Fuji and Aska-SS; Fig. 4A; Supplementary Fig. S4A). In addition, the simultaneous depletion of *CREBBP* and *EP300* downregulated the expression of *KREMEN2* mRNA in SMARCA4/SMARCA2-deficient cells and SS18–SSX fusion cells (Fig. 4B). These results indicate that CBP and p300 are involved in transcriptional upregulation of the *KREMEN2* gene in SMARCA4/SMARCA2-deficient and SS18–SSX fusion cells.

KREMEN2 also plays a role in suppressing apoptosis (34). Previously, we showed that downregulation of *KREMEN2* via dual inhibition of CBP/p300 in SMARCB1-deficient cells upregulated a proapoptotic marker gene *CASP6* and induced apoptosis (19). Thus, we examined whether downregulating *KREMEN2* via dual inhibition of CBP/p300 in SMARCA4/SMARCA2-deficient cells and SS18–SSX fusion cells induces apoptosis. Treatment with CBP/p300 dual inhibitors CP-C27 and A-485 induced apoptosis in SMARCA4/SMARCA2-deficient DMS114 cells and SS18–SSX fusion Fuji cells, as manifested by an increase in the number of cells that were positive for the apoptosis marker annexin V (Fig. 4C; Supplementary Fig. S4B). In addition, treatment of SMARCA4/SMARCA2-deficient cells and SS18–SSX fusion cells with CP-C27 and A-485 increased expression of the proapoptotic marker gene *CASP6* (Fig. 4D; Supplementary Fig. S4C). The *CASP6* gene in SMARCA4/SMARCA2-deficient cells and SS18–SSX fusion cells was upregulated by simultaneous knockdown of *CREBBP and EP300* (Fig. 4E); thus, these results indicate that simultaneous inhibition of CBP/p300 in SMARCA4/SMARCA2-deficient cells and SS18–SSX fusion cells induces apoptosis.

To confirm the involvement of KREMEN2 in suppressing apoptosis in SMARCA4/SMARCA2-deficient cells and SS18–SSX fusion cells, we investigated whether the knockdown of *KREMEN2* alters the expression of *CASP6*. Expression of the *CASP6* gene in SMARCA4/SMARCA2-deficient cells and SS18–SSX fusion cells was upregulated by knockdown of *KREMEN2* (Fig. 4F), indicating that repression of *KREMEN2* in SMARCA4/SMARCA2-deficient cells and SS18–SSX fusion cells induces apoptosis. To further confirm that KREMEN2 is a determinant of synthetic lethality through simultaneous inhibition of CBP/p300 in SMARCA4/SMARCA2-deficient cells and SS18–SSX fusion cancer cells, we investigated whether depleting *KREMEN2* affects cell viability. Depletion of *KREMEN2* (Supplementary Fig. S4D) reduced the viability of SMARCA4/SMARCA2-deficient cells and SS18–SSX fusion cells but not that of SWI/SNF-proficient cells (Fig. 4G). Thus, SMARCA4/SMARCA2-deficient and SS18–SSX fusion cancer cells depend on the expression of *KREMEN2*. These results indicate that inhibiting CBP/p300 in SMARCA4/SMARCA2-deficient cells and SS18–SSX fusion cells induces apoptosis by downregulating *KREMEN2*.

### Downregulation of *KREMEN2* in SMARCA4/SMARCA2-deficient and SS18–SSX fusion cancer cells through CBP/p300 inhibition induces apoptosis via KREMEN1

**Figure 5:**
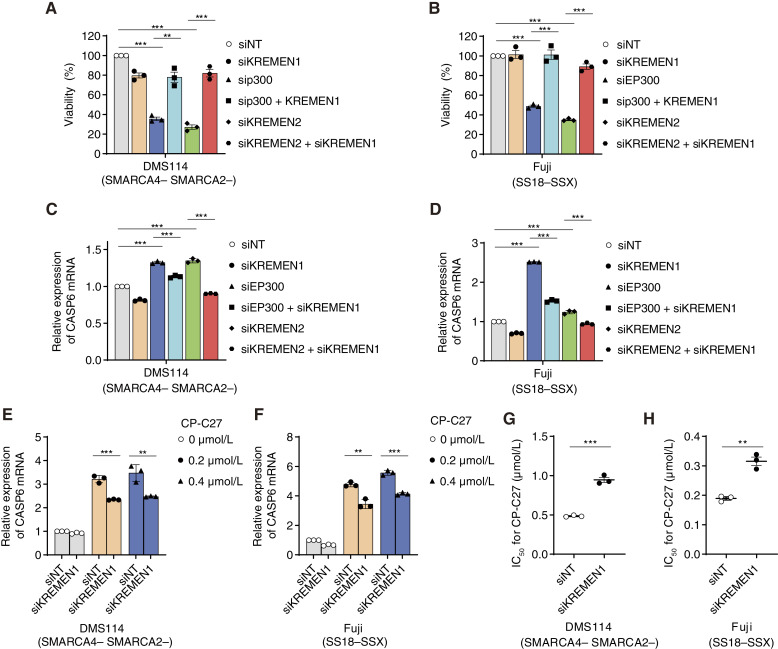
Downregulation of *KREMEN2* through CBP/p300 inhibition in SMARCA4/SMARCA2-deficient and SS18–SSX fusion cancer cells induces apoptosis via KREMEN1. **A** and **B,** Viability of SMARCA4/SMARCA2-deficient DMS114 (**A**) and SS18–SSX fusion Fuji (**B**) cell lines transfected with the indicated siRNAs. Cells were transfected with indicated siRNAs for 48 hours, reseeded, and transfected with the indicated siRNAs for an additional 48 hours. The cells were then reseeded again and incubated for 7 days. Data are presented as the mean ± SEM; *n* = 3 independent experiments. **C** and **D,** Expression of *CASP6* mRNA (relative to that in siNT-transfected cells) in SMARCA4/SMARCA2-deficient DMS114 (**C**) and SS18–SSX fusion Fuji (**D**) cell lines transfected with the indicated siRNAs. Cells were transfected with indicated siRNAs for 96 hours. Data are presented as the mean ± SD; *n* = 3 independent experiments. **E** and **F,** Expression of *CASP6* mRNA (relative to that in siNT-transfected cells) in SMARCA4/SMARCA2-deficient DMS114 (**E**) and SS18–SSX fusion Fuji (**F**) cell lines transfected with the indicated siRNAs. Cells were transfected with indicated siRNAs for 48 hours, reseeded, and transfected with the indicated siRNAs for 24 hours. The cells were then reseeded again and incubated for 24 hours. Data are presented as the mean ± SD; *n* = 3 independent experiments. **G** and **H,** IC_50_ values for CP-C27 in SMARCA4/SMARCA2-deficient DMS114 (**G**) and SS18–SSX fusion Fuji (**H**) cell lines transfected with the indicated siRNAs and treated with CBP/p300 inhibitor CP-C27. Cells were transfected for 48 hours with the indicated siRNAs. Cells were then transfected with the indicated siRNAs for an additional 24 hours. The cells were then reseeded again and treated for 4 days with CP-C27, and IC_50_ values were calculated based on cell viability. Data are presented as the mean ± SEM; *n* = 3 independent experiments. For all experiments, *P* values were determined by an unpaired two-tailed Student *t* test. *, *P* < 0.05; **, *P* < 0.01; ***, *P* < 0.001.

Like KREMEN2, KREMEN1 is a single-pass transmembrane protein interacting with KREMEN2 (34). KREMEN1 is involved in apoptosis induction (35), whereas KREMEN2 suppresses KREMEN1-mediated apoptosis (34). Previously, we demonstrated that downregulating *KREMEN2* by inhibiting CBP/p300 in SMARCB1-deficient cells induces apoptosis via KREMEN1 (19); therefore, we next assessed whether downregulating *KREMEN2* by inhibiting CBP/p300 induces apoptosis via KREMEN1 in SMARCA4/SMARCA2-deficient cells and SS18–SSX fusion cells and whether additional depletion of *KREMEN1* would inhibit apoptosis. First, we assessed whether synthetic lethality in SMARCA4/SMARCA2-deficient cells and SS18–SSX fusion cells caused by suppression of CBP/p300 could be prevented by depleting *KREMEN1*. Depleting *EP300* decreased the viability of SMARCA4/SMARCA2-deficient DMS114 cells (Fig. 5A; Supplementary Fig. S5A; Lane 3) and SS18–SSX fusion Fuji cells (Fig. 5B; Supplementary Fig. S5B; Lane 3); however, additional depletion of *KREMEN1* rescued the viability of SMARCA4/SMARCA2-deficient DMS114 cells (Fig. 5A; Supplementary Fig. S5C; Lane 4) and SS18–SSX fusion Fuji cells (Fig. 5B; Supplementary Fig. S5D; Lane 4), indicating that KREMEN1 mediates the decrease in cell viability induced by depleting *EP300*. Next, we investigated whether synthetic lethality caused by *KREMEN2* depletion in SMARCA4/SMARCA2-deficient cells and SS18–SSX fusion cells is prevented by additional depletion of *KREMEN1*. Depletion of *KREMEN2* decreased the viability of SMARCA4/SMARCA2-deficient DMS114 cells (Fig. 5A; Supplementary Fig. S5E; Lane 5) and SS18–SSX fusion Fuji cells (Fig. 5B; Supplementary Fig. S5F; Lane 5); however, additional depletion of *KREMEN1* rescued the viability of SMARCA4/SMARCA2-deficient DMS114 cells (Fig. 5A; Supplementary Fig. S5C; Lane 6) and SS18–SSX fusion Fuji cells (Fig. 5B; Supplementary Fig. S5D; Lane 6) significantly, indicating that KREMEN1 mediates the decrease in viability induced by depletion of *KREMEN2*. Correspondingly, upregulating *CASP6* by depleting *EP300* (Fig. 5C and D; Lane 3) or *KREMEN2* (Fig. 5C and D; Lane 5) from SMARCA4/SMARCA2-deficient DMS114 cells and SS18–SSX fusion Fuji cells was prevented by additional depletion of *KREMEN1* (Fig. 5C and D; lanes 4 and 6). In addition, upregulating *CASP6* in SMARCA4/SMARCA2-deficient cells and SS18–SSX fusion cells by a CBP/p300 dual inhibitor was also prevented by depletion of *KREMEN1* (Fig. 5E and F). Concordantly, sensitivity to a CBP/p300 dual inhibitor in SMARCA4/SMARCA2-deficient cells and SS18–SSX fusion cells were rescued by depletion of *KREMEN1* (Fig. 5G and H). These results indicate that downregulating *KREMEN2* by inhibiting CBP/p300 in SMARCB1-deficient cells triggers apoptosis mediated by KREMEN1.

### Treatment with a CBP/p300 dual inhibitor suppresses the growth of tumor xenografts derived from SMARCA4/SMARCA2-deficient cells and SS18–SSX fusion cells

**Figure 6:**
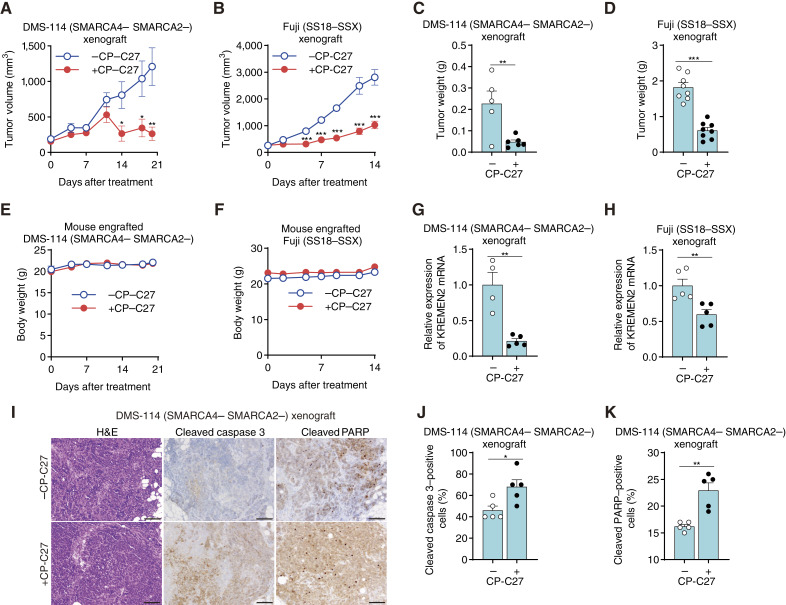
A CBP/p300 dual inhibitor suppresses the growth of tumor xenografts derived from SMARCA4/SMARCA2-deficient cells and SS18–SSX fusion cells. **A,** Volume of tumor xenografts derived from SMARCA4/SMARCA2-deficient DMS114 cell lines and harvested from mice treated once daily with 30 mg/kg CP-C27. Data are presented as the mean ± SEM. −CP-C27 (*n* = 5 biologically independent mice per group); +CP-C27 (*n* = 6 biologically independent mice per group). **B,** Volume of tumor xenografts derived from SS18–SSX fusion Fuji cell lines and harvested from mice treated once daily with 30 mg/kg CP-C27. Data are presented as the mean ± SEM. −CP-C27 (*n* = 8 biologically independent mice per group); +CP-C27 (*n* = 8 biologically independent mice per group). **C,** Weight of tumor xenografts derived from SMARCA4/SMARCA2-deficient DMS114 cell lines and harvested from mice treated once daily with 30 mg/kg CP-C27. Data are presented as the mean ± SEM. −CP-C27 (*n* = 5 biologically independent mice per group); +CP-C27 (*n* = 6 biologically independent mice per group). **D,** Weight of tumor xenografts derived from SS18–SSX fusion Fuji cell lines and harvested from mice treated once daily with 30 mg/kg CP-C27. Data are presented as the mean ± SEM. −CP-C27 (*n* = 8 biologically independent mice per group); +CP-C27 (*n* = 8 biologically independent mice per group). **E,** Body weight of mice engrafted with SMARCA4/SMARCA2-deficient DMS114 cell lines and treated once daily with 30 mg/kg CP-C27. Data are presented as the mean ± SEM. −CP-C27 (*n* = 5 biologically independent mice per group); +CP-C27 (*n* = 6 biologically independent mice per group). **F,** Body weight of mice engrafted SS18–SSX fusion Fuji cell lines and treated once daily with 30 mg/kg CP-C27. Data are presented as the mean ± SEM. −CP-C27 (*n* = 8 biologically independent mice per group); +CP-C27 (*n* = 8 biologically independent mice per group). **G,** Expression of *KREMEN2* mRNA (relative to the average of expression levels in −CP-C27 tumors) in xenografts derived from SMARCA4/SMARCA2-deficient DMS114 cell lines isolated from mice treated once daily with 30 mg/kg CP-C27. Data are presented as the mean ± SEM. −CP-C27 (*n* = 4 biologically independent mice per group); +CP-C27 (*n* = 5 biologically independent mice per group). **H,** Expression of *KREMEN2* mRNA (relative to the average of expression levels in −CP-C27 tumors) in xenografts derived from SS18–SSX fusion Fuji cell lines isolated from mice treated once daily with 30 mg/kg CP-C27. Data are presented as the mean ± SEM. −CP-C27 (*n* = 5 biologically independent mice per group); +CP-C27 (*n* = 5 biologically independent mice per group). **I,** Representative immunohistochemical staining of cleaved PARP and cleaved caspase-3 in xenografts derived from SMARCA4/SMARCA2-deficient DMS114 cell lines and isolated from mice treated once daily for 20 days with 30 mg/kg CP-C27: Scale bar, 100 μm. **J** and **K,** Percentage of cells positive for cleaved caspase-3 (**J**) and cleaved PARP (**K**) in xenografts derived from SMARCA4/SMARCA2-deficient DMS114 cell lines and isolated from mice treated once daily for 20 days with 30 mg/kg CP-C27. Data are presented as the mean ± SEM. −CP-C27 (*n* = 5 biologically independent mice per group); +CP-C27 (*n* = 5 biologically independent mice per group). For all experiments, *P* values were determined by an unpaired two-tailed Student *t* test. *, *P* < 0.05; **, *P* < 0.01; ***, *P* < 0.001. H&E, hematoxylin and eosin.

Finally, to investigate the effects of the CBP/p300 dual inhibitor CP-C27 *in vivo*, we used it to treat mice bearing s.c. xenografts. Twice-daily treatment with 50 mg/kg of CP-C27 led to significant suppression of tumors derived from SMARCA4/SMARCA2-deficient A427 and TOV112D cells (Supplementary Fig. S6A–S6D). Importantly, none of these doses had any adverse effect on the body weight of mice (Supplementary Fig. S6E and S6F). In addition, the concentrations of CP-C27 in the xenograft tumors were maintained at >1 μmol/L, whereas those in plasma decreased to <1 μmol/L (Supplementary Fig. S6G). We also confirmed the attenuation of H3K27ac in xenografts treated with the CBP/p300 dual inhibitor CP-C27 (Supplementary Fig. S6H and S6I). Moreover, once-daily treatment with CP-C27 led to marked suppression of tumor growth in SMARCA4/SMARCA2-deficient DMS114 and SS18–SSX fusion Fuji xenograft models (Fig. 6A–D), again with no adverse effect on the body weight of mice (Fig. 6E and F). CP-C27 attenuated expression of the *KREMEN2* gene in the SMARCA4/SMARCA2-deficient DMS114 and SS18–SSX fusion Fuji xenograft models (Fig. 6G and H). Treatment of SMARCA4/SMARCA2-deficient DMS114 xenografts with CP-C27 led to a significant increase in the number of cells positive for the apoptotic markers cleaved caspase-3 and cleaved PARP (Fig. 6I–K). These *in vivo* observations support the results obtained from the cell line models and indicate that a CBP/p300 inhibitor suppresses the growth of SMARCA4/SMARCA2-deficient and SS18–SSX fusion xenograft tumors.

## Discussion

The principle of synthetic lethality is based on the “one-on-one” relationship between two genes, gene *A* and gene *B* (3, 36). In this study, we show that simultaneous inhibition of a paralog pair (CBP and p300) causes synthetic lethality in SMARCA4/SMARCA2-deficient cancer cells and SS18–SSX fusion cancer cells; these results are similar to those obtained for SMARCB1-deficient cancer cells (19). This concept illustrates the “two-on-one” relationship between two genes and another gene. Importantly, targeting a paralog pair rather than two different targets has several advantages because paralog proteins are so similar that a single agent can inhibit both. In contrast, two inhibitors are required to inhibit two distinct proteins. Thus, it should be possible to develop advanced synthetic lethal therapies that inhibit both proteins in a paralog pair within a cancer cell harboring a gene mutation; we call the screening method “simultaneous inhibition of a paralog pair as a synthetic lethal target.” Using this method, we show in this study that simultaneous inhibition of *CREBB*/*EP300* causes synthetic lethality in cancer cells not only harboring a single gene deficiency (i.e., SMARCB1-deficient; ref. 19) but also in cells harboring a deficiency in two genes (i.e., SMARCA4/SMARCA2-deficient) or a gene fusion (i.e., SS18–SSX fusion gene).

Strategies for cancer treatment based on the synthetic lethality between paralogs targeting one paralog in cancers with a mutation of another paralog have been proposed by many groups (10, 37–45). In addition, several groups have reported attempts to search for synthetic lethal paralog pairs genome-wide from about 400 to 1,030 paralog pairs using the Cas9 or Cas12a CRISPR knockout system (46–49). In these studies, only about 3% to 4% of paralog pairs were essential for cell growth when paralog pairs were suppressed simultaneously. In addition, studies that integrated analyses of these study data have shown similar results (50). Because it does not show lethality even if most paralog pairs are inhibited simultaneously, it is expected that the possibility of side effects may be slight in the development of simultaneous paralog pair inhibitors. Taking advantage of these points, we applied the “simultaneous inhibition of a paralog pair as a synthetic lethal target” method as a strategy to identify synthetic lethal targets for cancers with defective genetic abnormalities. Furthermore, by using a “simultaneous inhibition of a paralog pair as a synthetic lethal target” method in a genome-wide manner, it will be possible to identify new targets that could not be found by the search method for synthetic lethal targets by inhibition of single factors that have been explored by the database of the DepMap project (51, 52).

SWI/SNF chromatin remodeling complexes can be classified broadly into subcomplexes cBAF, PBAF, and ncBAF (6). SMARCB1 is included in cBAF and PBAF. Either SMARCA4 or SMARCA2 are included in all cBAF, PBAF, and ncBAF complexes, albeit mutually exclusive. SS18 is included in cBAF and ncBAF. In addition to SMARCB1 deficiency (19), cells deficient in SMARCA4/SMARCA2 and cells with an SS18–SSX fusion show increased expression of *KREMEN2*; simultaneous inhibition of CBP/p300 in these cells represses *KREMEN2* expression and induces cell death. Thus, which subcomplexes within the SWI/SNF complex regulate expression of the *KREMEN2* and which determines sensitivity to CBP/p300 dual inhibitors? In the present study, we found that expression of *KREMEN2* increased in cells deficient in both SMARCA4 and SMARCA2 contained within cBAF, PBAF, and ncBAF. When SS18–SSX is incorporated into cBAF but not ncBAF, the cBAF subcomplex collapses (32); thus, SS18–SSX fusion cells can be considered deficient in the cBAF subcomplex. This indicates that *KREMEN2* expression could be increased by cBAF deficiency induced by SS18–SSX fusion. The subcomplex commonly associated with increased sensitivity to CBP/p300 dual inhibitors, as well as increased expression of *KREMEN2* in cells with SMARCB1 deficiency (cBAF and PBAF deficiency), SMARCA4/SMARCA2 deficiency (cBAF, PBAF, and ncBAF deficiency), or SS18–SSX fusion (cBAF deficiency), is the cBAF subcomplex. In other words, increased expression of *KREMEN2* in cancers deficient in the cBAF complex has antiapoptotic effects, and repression of *KREMEN2* via inhibition of CBP/p300 is the determinant of synthetic lethality. Our previous study showed that cBAF localized at the promoter region of the *KREMEN2* locus (19). SMARCA4 and ARID1A are contained in the cBAF subcomplex. The sensitivity of SMARCA4-deficient cancer cell lines to CBP/p300 dual inhibitors was similar to that of SWI/SNF WT cell lines. On the other hand, the sensitivity of ARID1A-deficient cancer cell lines to CBP/p300 dual inhibitors was intermediate between SWI/SNF WTs and the sensitive genotypes (SMARCA4/SMARCA2-deficient and SS18–SSX fusion). A deficiency of SMARCA4 will not affect the function of the cBAF subcomplex, including SMARCA2. On the other hand, a deficiency of ARID1A may affect the function of both the cBAF subcomplex containing SMARCA4 and the cBAF subcomplex containing SMARCA2. These suggest that the absence of ARID1A has a more significant effect on the cBAF subcomplex than the deficiency of only SMARCA4, which may cause sensitivity to simultaneous CBP/p300 inhibitors.

Taken together, the data in the previous (19) and current studies suggest that the molecular mechanism underlying induction of synthetic lethality by simultaneous inhibition of CBP/p300 in cBAF-deficient cancers is as follows: localization of the cBAF complexes to the promotor region of the *KREMEN2* gene locus leads to transcriptional repression of the *KREMEN2* gene. cBAF deficiency increases the expression of *KREMEN2* by allowing CBP/p300 to localize at the promoter region. In cBAF-deficient cancer cells, inhibition of CBP/p300 leads to marked repression of *KREMEN2*, which reduces cell viability; this suggests that expression of the *KREMEN2* gene is induced cooperatively by CBP and p300 and that it is required for the viability of cBAF-deficient cells. When expression of *KREMEN2* is downregulated by simultaneous inhibition of CBP/p300, monomerization of KREMEN1 induces apoptosis. The data show that downregulating *KREMEN2* in cBAF-deficient cells using CBP/p300 dual inhibitors induces apoptosis via monomerization of KREMEN1.

The aberrations of SMARCB1, SMARCA4, ARID1A, PBRM1, and SS18 within the SWI/SNF complex are frequently found in rare cancers and refractory cancers (3). This study examined the sensitivity of these representative constituent SWI/SNF genes to CBP/p300 dual inhibitors. We found that CBP/p300 dual inhibitors are not promising agents for all cancers harboring abnormal SWI/SNF complex constituents. However, they are effective against cancers that are completely deficient in the cBAF subcomplex. In other words, CBP/p300 dual inhibitors are a promising treatment for SMARCB1-deficient rhabdoid tumors (almost 100%), epithelioid sarcomas (nearly 100%), SMARCA4/SMARCA2-deficient non–small cell lung cancer (10%), ovarian small cell carcinoma (100%), and SS18–SSX fusion synovial sarcomas (100%; ref. 3). Non–small cell lung cancer harboring a SMARCA4/SMARCA2 deficiency has no established treatment because it is mutually exclusive to genetic abnormalities such as EGFR mutations and anaplastic lymphoma kinase (ALK) and RET fusions, for which there are established treatments (53). In addition, rhabdoid tumors, epithelioid sarcoma, ovarian small cell carcinoma, and synovial sarcoma are rare cancers for which promising therapies have not yet been established. Thus, CBP/p300 simultaneous inhibitors are expected to lead to the establishment of promising treatments for such cancers.

## Supplementary Material

Supplementary Figure 1 CBP and p300 dual inhibitors selectively sensitizes cancer cells deficient in a subunit of cBAF complex.

Supplementary Figure 2 Simultaneous inhibition of CBP/p300 causes synthetic lethality in SMARCA4/SMARCA2-deficient and SS18-SSX-fusion cells.

Supplementary Figure 3 SMARCA4/SMARCA2-deficiency and SS18-SSX-fusion lead to upregulation of KREMEN2 gene expression.

Supplementary Figure 4 Simultaneous inhibition of CBP/p300 in SMARCB1-deficient cells induces synthetic lethality by downregulating KREMEN2.

Supplementary Figure 5 Downregulation of KREMEN2 through CBP/p300 inhibition in SMARCA4/SMARCA2-deficient and SS18-SSX-fusion cancer cells induces apoptosis via KREMEN1.

Supplementary Figure 6 A CBP/p300 dual inhibitor suppresses growth of tumor xenografts derived from SMARCA4/SMARCA2-deficient cells and SS18-SSX-fusion cells.
