# Supplementary material for: Efficacy of CBP/p300 Dual Inhibitors against Derepression of KREMEN2 in cBAF-Deficient Cancers
Source: Cancer Res Commun. 2025 Jan 6;5(1):24–38. doi: 10.1158/2767-9764.CRC-24-0484 (PMC11701801; doi:10.1158/2767-9764.CRC-24-0484)
Supplement: Supplementary Figure 1 — CBP and p300 dual inhibitors selectively sensitizes cancer cells deficient in a subunit of cBAF complex. [file crc-24-0484_supplementary_figure_1_suppsf1.pdf]

Supplementary Figure 1

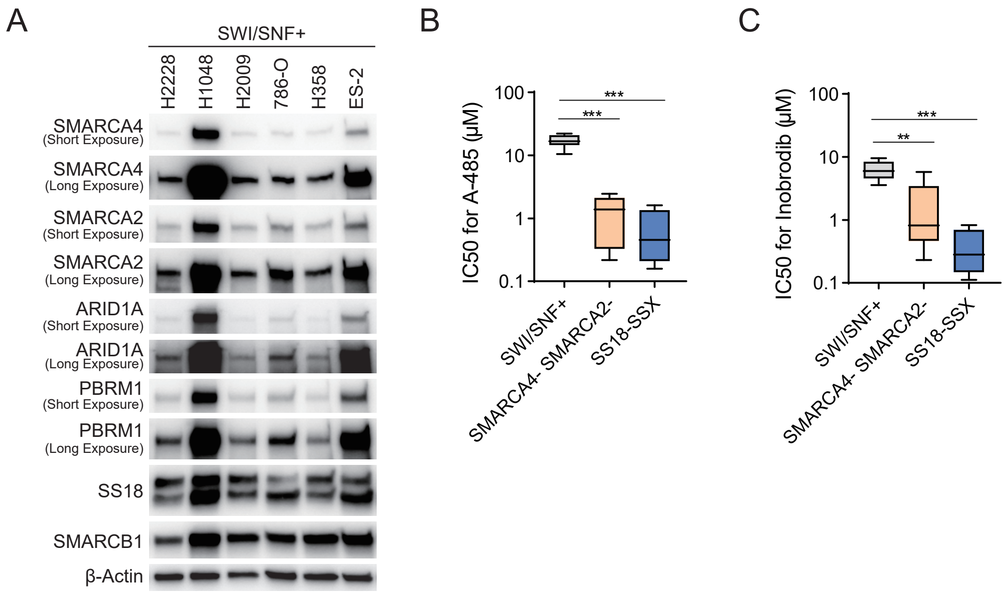

Supplementary Figure 1. CBP and p300 dual inhibitors selectively sensitizes cancer cells deficient in a subunit of cBAF complex.
A. Immunoblot analysis of SMARCA4, SMARCA2, ARID1A. PBRM1, SS18, SMARCB1 and β-actin expression in SWI/SNF-proficient cell lines.
B, C. IC50 values for CBP/p300 inhibitors A-485 (**B**) and Inobrodib (**C**) in the SMARCB1-proficient (H2228, H1048, H2009, 786-O, H358, ES-2), SMARCA4-/SMARCA2-deficient (SBC-5, A427, SW13, DMS114, TOV112D), and SS18-SSX-fusion (Aska-SS, HS-SY-II, Yamato-SS, Fuji) cell line groups. Cells were treated with inhibitors for 6 days, and IC50 values were calculated based on cell viability. Data are presented as the mean ± SEM (standard error of the mean); SWI/SNF-proficient (n = 7), SMARCA4-/SMARCA2-deficient (n = 5), and SS18-SSX-fusion (n = 4).
For all experiments, p values were determined by an unpaired two-tailed Student's t-test. *$p < 0.05$, **$p < 0.01$, ***$p < 0.001$.
