# Supplementary material for: Efficacy of CBP/p300 Dual Inhibitors against Derepression of KREMEN2 in cBAF-Deficient Cancers
Source: Cancer Res Commun. 2025 Jan 6;5(1):24–38. doi: 10.1158/2767-9764.CRC-24-0484 (PMC11701801; doi:10.1158/2767-9764.CRC-24-0484)
Supplement: Supplementary Figure 2 — Simultaneous inhibition of CBP/p300 causes synthetic lethality in SMARCA4/SMARCA2-deficient and SS18-SSX-fusion cells. [file crc-24-0484_supplementary_figure_2_suppsf2.pdf]

Supplementary Figure 2

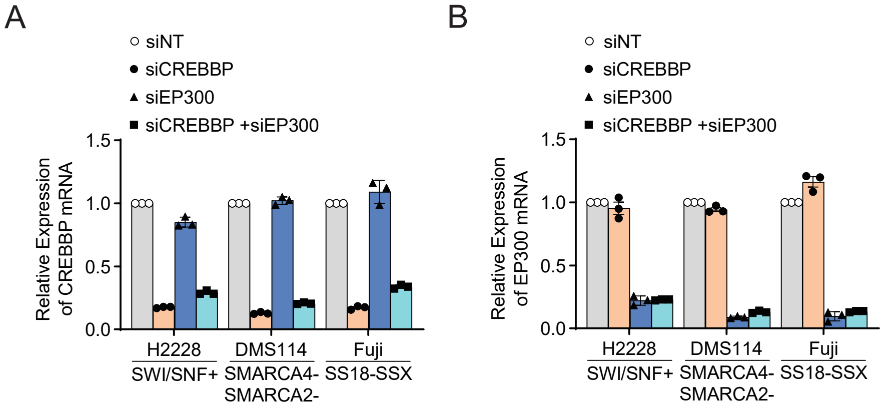

Supplementary Figure 2. Simultaneous inhibition of CBP/p300 causes synthetic lethality in SMARCA4/SMARCA2-deficient and SS18-SSX-fusion cells. **A, B,** Relative expression of *CREBBP* (**A**) and *EP300* (**B**) mRNA in SWI/SNF-proficient H2228, SMARCA4-/SMARCA2-deficient DMS114, and SS18-SSX-fusion Fuji cell lines transfected for 48 h with the indicated siRNAs. Data are presented as the mean ±SD (standard deviation); n = 3 independent experiments.
