# Supplementary material for: Efficacy of CBP/p300 Dual Inhibitors against Derepression of KREMEN2 in cBAF-Deficient Cancers
Source: Cancer Res Commun. 2025 Jan 6;5(1):24–38. doi: 10.1158/2767-9764.CRC-24-0484 (PMC11701801; doi:10.1158/2767-9764.CRC-24-0484)
Supplement: Supplementary Figure 3 — SMARCA4/SMARCA2-deficiency and SS18-SSX-fusion lead to upregulation of KREMEN2 gene expression. [file crc-24-0484_supplementary_figure_3_suppsf3.pdf]

Supplementary Figure 3

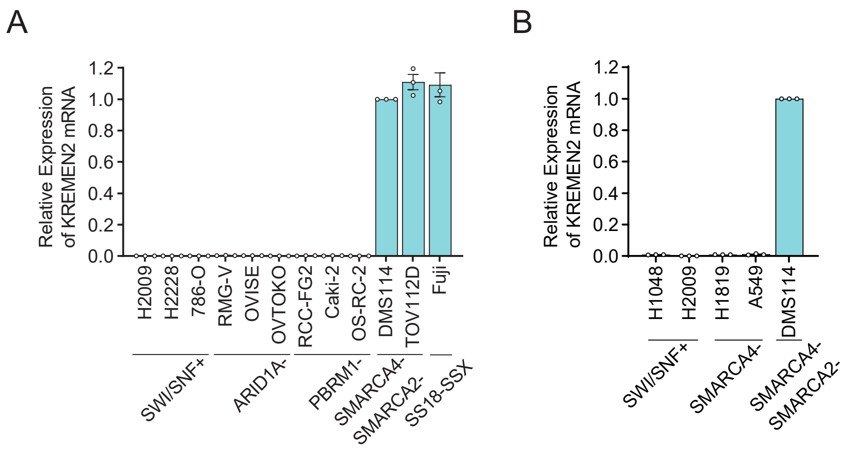

Supplementary Figure 3. SMARCA4/SMARCA2-deficiency and SS18-SSX-fusion lead to upregulation of *KREMEN2* gene expression.
A, Expression of *KREMEN2* mRNA in SWI/SNF-proficient, ARID1A-deficient, PBRM1-deficient, SMARCA4/SMARCA2-deficient, and SS18-SSX-fusion cell lines (relative to that in DMS-114 cells). Data are presented as the mean ± SEM (standard error of the mean); n = 3 independent experiments.
B, Expression of *KREMEN2* mRNA in SWI/SNF-proficient, SMARCA4-deficient, and SMARCA4/SMARCA2-deficient cell lines (relative to that in DMS114 cells). Data are presented as the mean ± SEM; n = 3 independent experiments.
