# Supplementary material for: Efficacy of CBP/p300 Dual Inhibitors against Derepression of KREMEN2 in cBAF-Deficient Cancers
Source: Cancer Res Commun. 2025 Jan 6;5(1):24–38. doi: 10.1158/2767-9764.CRC-24-0484 (PMC11701801; doi:10.1158/2767-9764.CRC-24-0484)
Supplement: Supplementary Figure 4 — Simultaneous inhibition of CBP/p300 in SMARCB1-deficient cells induces synthetic lethality by downregulating KREMEN2. [file crc-24-0484_supplementary_figure_4_suppsf4.pdf]

# Supplementary Figure 4

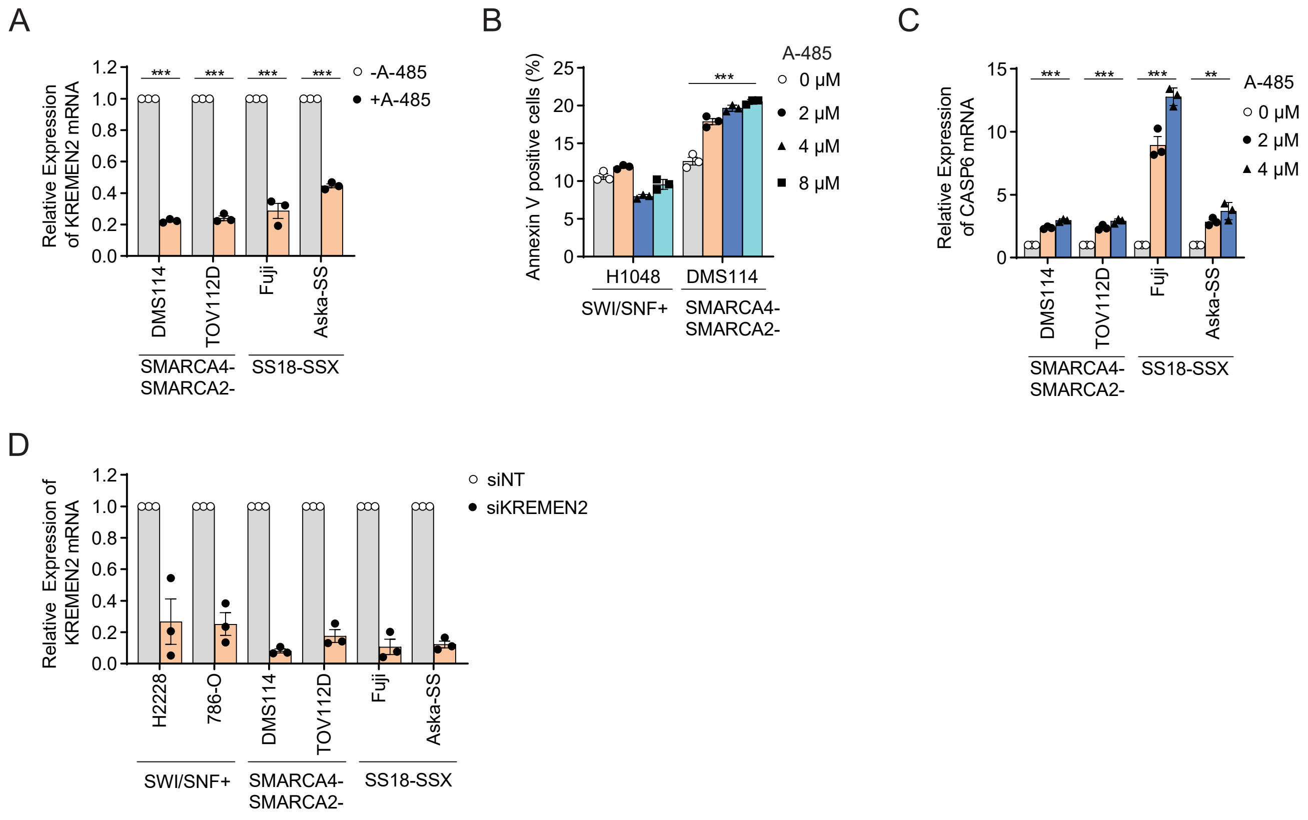


**Supplementary Figure 4.** Simultaneous inhibition of CBP/p300 in SMARCB1-deficient cells induces synthetic lethality by downregulating *KREMEN2*.

**A**, Expression of *KREMEN2* mRNA (relative to that in non-treated cells) in SMARCA4/SMARCA2-deficient and SS18-SSX-fusion cells treated for 24 h with the indicated concentration of A-485. Data are presented as the mean ± SD (standard deviation); n = 3 independent experiments.

**B**, Percentage of Annexin V-positive cells within the SWI/SNF-proficient H1048 and SMARCA4/SMARCA2-deficient DMS114 cell populations treated for 6 days with the indicated concentration of A-485. Data are presented as the mean ± SD; n = 3 independent experiments.

**C**, Expression of *CASP6* mRNA (relative to that in non-treated cells) in SMARCA4/SMARCA2-deficient and SS18-SSX-fusion cells treated with the indicated concentration of A-485 for 24 h. Data are presented as the mean ± SD, n = 3 independent experiments.

**D**, Expression of *KREMEN2* mRNA (relative to that in siNT-transfected cells) in SWI/SNF-proficient, SMARCA4/SMARCA2-deficient, and SS18-SSX-fusion cells transfected for 48 h with the indicated siRNAs. Data are presented as the mean ± SD; n = 3 independent experiments.

For all experiments, p values were determined by an unpaired two-tailed Student's t-test. *$p < 0.05$, **$p < 0.01$, ***$p < 0.001$.
