# Supplementary material for: Efficacy of CBP/p300 Dual Inhibitors against Derepression of KREMEN2 in cBAF-Deficient Cancers
Source: Cancer Res Commun. 2025 Jan 6;5(1):24–38. doi: 10.1158/2767-9764.CRC-24-0484 (PMC11701801; doi:10.1158/2767-9764.CRC-24-0484)
Supplement: Supplementary Figure 5 — Downregulation of KREMEN2 through CBP/p300 inhibition in SMARCA4/SMARCA2-deficient and SS18-SSX-fusion cancer cells induces apoptosis via KREMEN1. [file crc-24-0484_supplementary_figure_5_suppsf5.pdf]

# Supplementary Figure 5

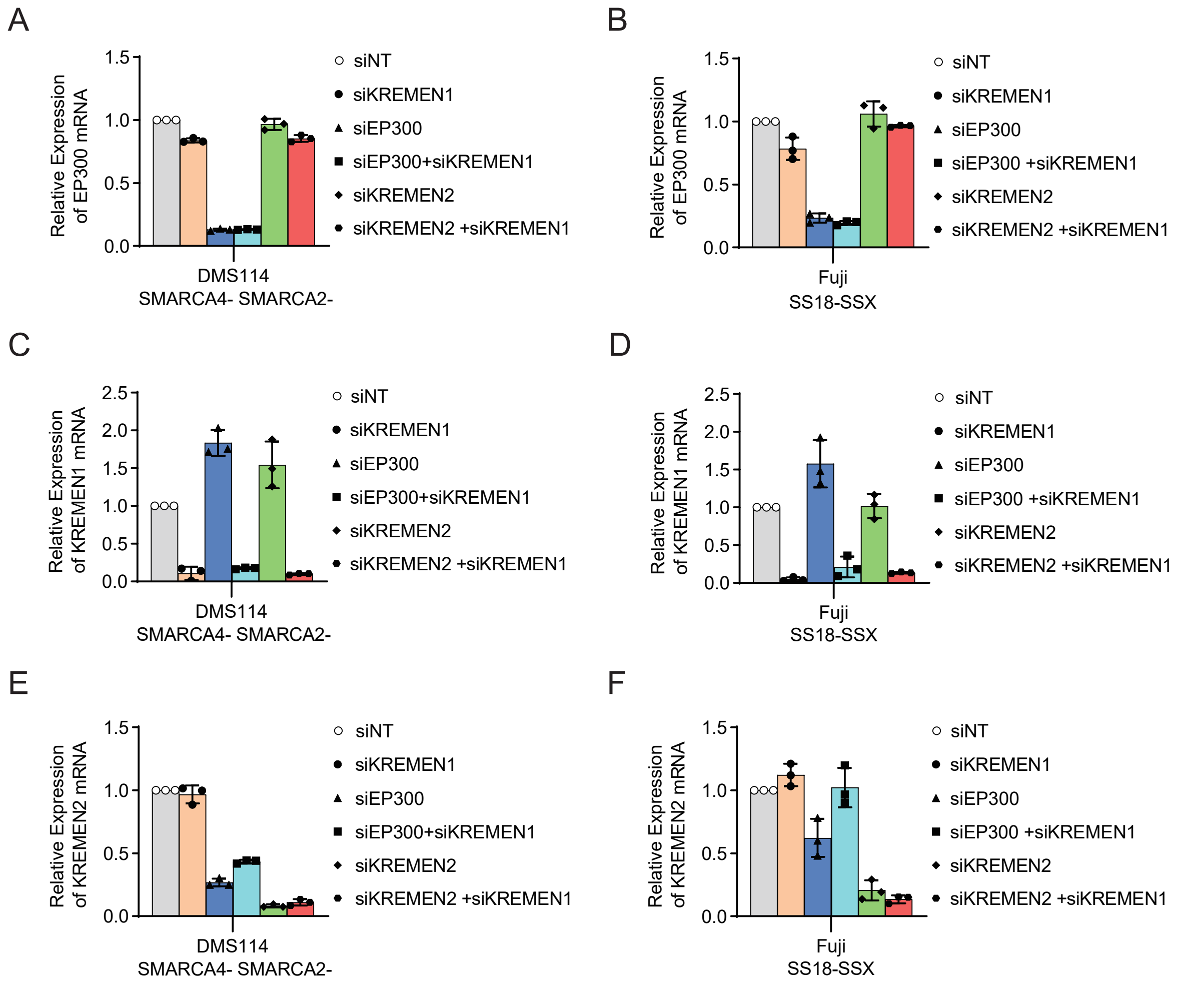


**Supplementary Figure 5**. Downregulation of *KREMEN2* through CBP/p300 inhibition in SMARCA4/SMARCA2-deficient and SS18-SSX-fusion cancer cells induces apoptosis via KREMEN1.

**A, B,** Expression of *EP300* mRNA (relative to that in siNT (non-targeting)-transfected cells) in SMARCA4/SMARCA2-deficient DMS114 (**A**) and SS18-SSX-fusion Fuji (**B**) cell lines transfected with the indicated siRNAs. Cells were transfected with the indicated siRNAs for 48 h. Data are presented as the mean ± SD (standard deviation); n = 3 independent experiments.

**C, D,** Expression of *KREMEN1* mRNA (relative to that in siNT (non-targeting)-transfected cells) in SMARCA4/SMARCA2-deficient DMS114 (**C**) and SS18-SSX-fusion Fuji (**D**) cell lines transfected with the indicated siRNAs. Cells were transfected with the indicated siRNAs for 48 h. Data are presented as the mean ± SD; n = 3 independent experiments.

**E, F,** Expression of *KREMEN2* mRNA (relative to that in siNT (non-targeting)-transfected cells) in SMARCA4/SMARCA2-deficient DMS114 (**E**) and SS18-SSX-fusion Fuji (**F**) cell lines transfected with the indicated siRNAs for 48 h. Data are presented as the mean ± SD; n = 3 independent experiments.
