# Supplementary material for: Efficacy of CBP/p300 Dual Inhibitors against Derepression of KREMEN2 in cBAF-Deficient Cancers
Source: Cancer Res Commun. 2025 Jan 6;5(1):24–38. doi: 10.1158/2767-9764.CRC-24-0484 (PMC11701801; doi:10.1158/2767-9764.CRC-24-0484)
Supplement: Supplementary Figure 6 — A CBP/p300 dual inhibitor suppresses growth of tumor xenografts derived from SMARCA4/SMARCA2-deficient cells and SS18-SSX-fusion cells. [file crc-24-0484_supplementary_figure_6_suppsf6.pdf]

Supplementary Figure 6

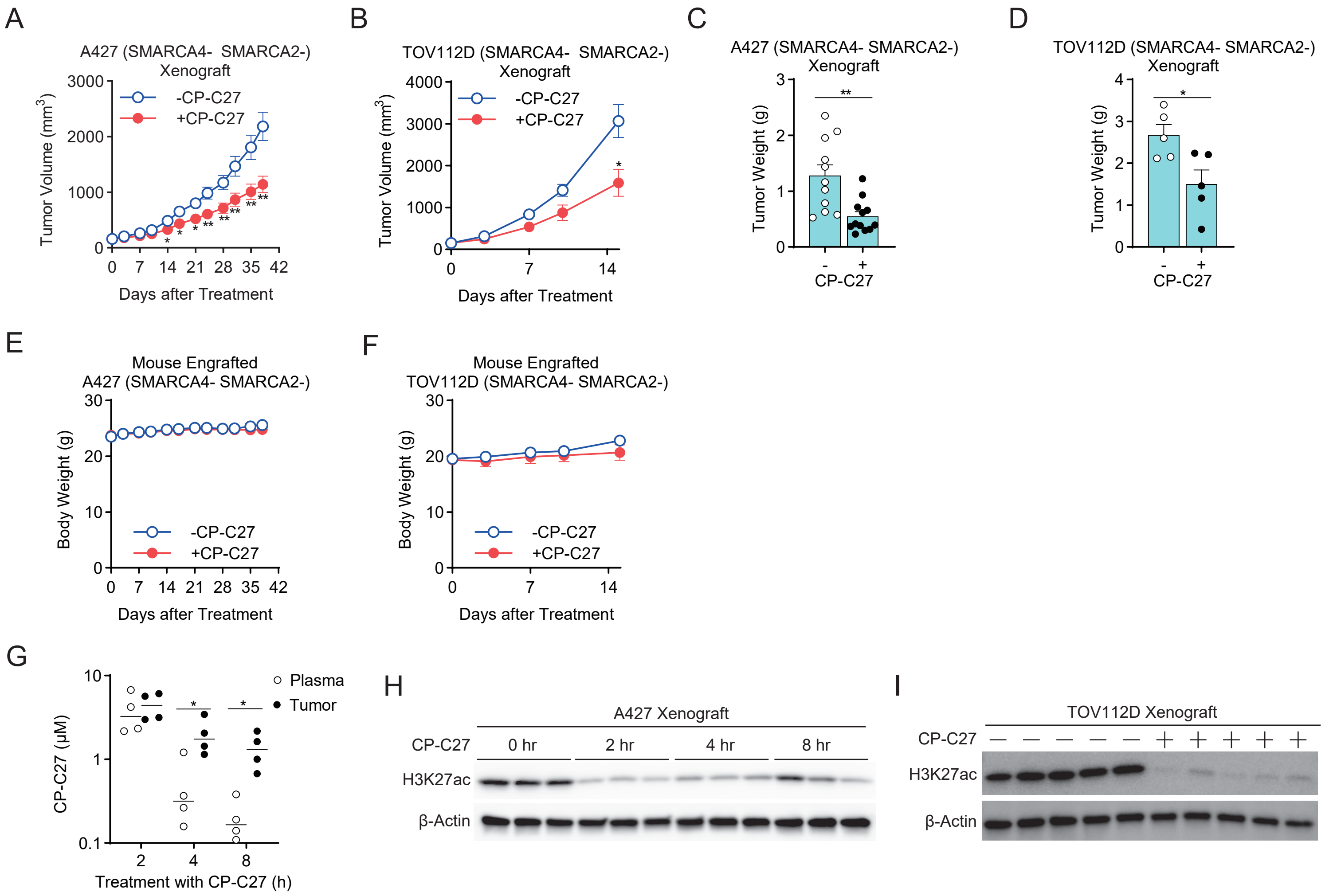

Supplementary Figure 6. A CBP/p300 dual inhibitor suppresses growth of tumor xenografts derived from SMARCA4/SMARCA2-deficient cells and SS18-SSX-fusion cells.

A, Volume of tumor xenografts derived from SMARCA4/SMARCA2-deficient A427 cell lines and harvested from mice treated twice daily with 50 mg/kg CP-C27. Data are presented as the mean ± SEM (standard error of the mean). -CP-C27 (n = 11 biologically independent mice per group); +CP-C27 (n = 12 biologically independent mice per group).

B, Volume of tumor xenografts derived from SMARCA4/SMARCA2-deficient TOV112D cell lines and harvested from mice treated twice daily with 50 mg/kg CP-C27. Data are presented as the mean ± SEM. -CP-C27 (n = 5 biologically independent mice per group); +CP-C27 (n = 5 biologically independent mice per group).

C, Weight of tumor xenografts derived from SMARCA4/SMARCA2-deficient A427 cell lines and harvested from mice treated twice daily with 50 mg/kg CP-C27. Data are presented as the mean ± SEM. -CP-C27 (n = 11 biologically independent mice per group); +CP-C27 (n = 12 biologically independent mice per group).

D, Weight of tumor xenografts derived from SMARCA4/SMARCA2-deficient TOV112D cell lines and harvested from mice treated twice daily with 50 mg/kg CP-C27. Data are presented as the mean ± SEM. -CP-C27 (n = 5 biologically independent mice per group); +CP-C27 (n = 5 biologically independent mice per group).

E, Body weight of mice engrafted SMARCA4/SMARCA2-deficient A427 cell lines treated twice daily with 50 mg/kg CP-C27. Data are presented as the mean ± SEM. -CP-C27 (n = 11 biologically independent mice per group); +CP-C27 (n = 12 biologically independent mice per group).

F, Body weight of mice engrafted SMARCA4/SMARCA2-deficient TOV112D cell lines treated twice daily with 50 mg/kg CP-C27. Data are presented as the mean ± SEM. -CP-C27 (n = 5 biologically independent mice per group); +CP-C27 (n = 5 biologically independent mice per group).

G, Concentration of CP-C27 in plasma and tumor xenografts derived from SMARCA4/SMARCA2-deficient A427 cell lines obtained from mice treated for the indicated times with 50 mg/kg CP-C27. Data are presented as the mean ± SEM; n = 4 biologically independent mice per group.

H, I, Immunoblot analysis of H3K27ac and β-actin expression in tumor xenografts derived from SMARCA4/SMARCA2-deficient A427 (H) and TOV112D (I) cell lines isolated from mice treated for the indicated times (**H**), and for 8 h (**I**) with 50 mg/kg CP-C27.

For all experiments, p values were determined by an unpaired two-tailed Student's t-test. *$p < 0.05$, **$p < 0.01$, ***$p < 0.001$.
